# Potential Dissociative Glucocorticoid Receptor Activity for Protopanaxadiol and Protopanaxatriol

**DOI:** 10.3390/ijms20010094

**Published:** 2018-12-27

**Authors:** Aikaterini G. Karra, Maria Konstantinou, Maria Tzortziou, Ioannis Tsialtas, Foteini D. Kalousi, Constantine Garagounis, Joseph M. Hayes, Anna-Maria G. Psarra

**Affiliations:** 1 Department of Biochemistry and Biotechnology, University of Thessaly, 41500 Larissa, Greece; Aikaterini.g.karra@gmail.com (A.G.K.); maira_kman_91@hotmail.com (M.K.); matziort@bio.uth.gr (M.T.) itsialtas@bio.uth.gr (I.T.); fokalous@bio.uth.gr (F.D.K.); constantine.garagounis@gmail.com (C.G.); 2 School of Pharmacy & Biomedical Sciences, University of Central Lancashire, Preston PR12HE, UK; JHayes@uclan.ac.uk

**Keywords:** protopanaxadiol, protopanaxatriol, glucocorticoid receptor, SEGRA, apoptosis, inflammation, ginsenosides, tranactivation, tranrepression

## Abstract

Glucocorticoids are steroid hormones that regulate inflammation, growth, metabolism, and apoptosis via their cognate receptor, the glucocorticoid receptor (GR). GR, acting mainly as a transcription factor, activates or represses the expression of a large number of target genes, among them, many genes of anti-inflammatory and pro-inflammatory molecules, respectively. Transrepression activity of glucocorticoids also accounts for their anti-inflammatory activity, rendering them the most widely prescribed drug in medicine. However, chronic and high-dose use of glucocorticoids is accompanied with many undesirable side effects, attributed predominantly to GR transactivation activity. Thus, there is a high need for selective GR agonist, capable of dissociating transrepression from transactivation activity. Protopanaxadiol and protopanaxatriol are triterpenoids that share structural and functional similarities with glucocorticoids. The molecular mechanism of their actions is unclear. In this study applying induced-fit docking analysis, luciferase assay, immunofluorescence, and Western blot analysis, we showed that protopanaxadiol and more effectively protopanaxatriol are capable of binding to GR to activate its nuclear translocation, and to suppress the nuclear factor-kappa beta activity in GR-positive HeLa and HEK293 cells, but not in GR-low level COS-7 cells. Interestingly, no transactivation activity was observed, whereas suppression of the dexamethasone-induced transactivation of GR and induction of apoptosis in HeLa and HepG2 cells were observed. Thus, our results indicate that protopanaxadiol and protopanaxatriol could be considered as potent and selective GR agonist.

## 1. Introduction

Glucocorticoids (GCs) are steroid hormones which exert their actions via binding to their cognate receptors, the glucocorticoid receptors (GRs). Glucocorticoid receptors belong to the large superfamily of nuclear receptors that act mainly as transcription factors [1]. GCs control many cellular functions such as metabolism, growth, development, immune and stress response, apoptosis, and glucose homeostasis [2]. The glucocorticoid receptor alpha (GR) is the most prominent transcriptionally active isoform of GRs. GR is predominantly localized in the cytoplasm. Upon ligand binding, GR is translocated into the nucleus where it positively or negatively regulates the transcription of a plethora of GR target genes, either by direct binding to specific hormone response elements of the nuclear DNA, or by interaction with other transcription factors and regulation of their target genes expression. GCs can also act by nongenomic, mechanisms of action, via activation of cytosolic or membrane-bound GR, such as membranous G-coupled glucocorticoid receptor, or via nonspecific physicochemical interactions with cell membrane [3,4,5,6]. Mitochondrial glucocorticoid receptor localization and direct or indirect regulation of mitochondrial actions via GCs are also documented [7,8,9,10].

Due to the crucial role of GCs in the regulation of human physiology and especially due to their strong anti-inflammatory, immunosuppressive and tissue-specific apoptotic activities, GCs are widely used for the treatment of acute and chronic inflammatory diseases, autoimmune diseases [11,12], as well as for the treatment of many oncology disorders such as hematopoietic malignancies of the lymphoid lineage [13,14]. The anti-inflammatory and immunosuppressive effects of GCs are exerted via (a) the activation of GCs transrepression activities, which are mediated by the negative glucocorticoid response element (GRE) dependent regulation of pro-inflammatory GR target genes expression, (b) via interaction of GR with other transcriptions factors such as Nuclear factor-kappa beta (NF-κΒ) and Signal Transducer and Activator of Transcription-3 (Stat3) and regulation of their actions, and (c) via activation of GRE–dependent transcription of many anti-inflammatory molecules [15,16].

On the other hand, the supra-physiological doses and chronic use of exogenous GCs, for medical purposes, often gives rise to many undesirable side effects, including diabetes induction, muscle wasting, and fat redistribution, effects associated with the GCs-induced gluconeogenesis and activation of catabolic actions such as lipolysis and protein degradation [17,18,19]. These actions are mostly mediated by GR transactivation, whereas GR transrepression is responsible for the side effect of hypothalamic pituitary axis (HPA) suppression, observed by steroids treatment. In addition, other side effects such as osteoporosis are mediated by both transactivation and transrepression [20].

Thus, there is a high need for potent selective steroidal or non-steroidal glucocorticoid receptor agonist (SEGRAs) or modulators (SEGRMs) that possess the desirable anti-inflammatory, immunosuppressive or anti-cancer activities of the classic GCs, but exert no or reduced side effects [20,21,22,23,24]. These compounds can bind to the same or to different GCs binding sites in the ligand binding domain (LBD) of GR. Binding of GR with these molecules may cause different GR conformational changes, affecting GR binding to DNA and/or recruitment of corepressors or coactivators in the GR-ligand complex, leading to modulation of GR functions [20].

**Figure 1:**
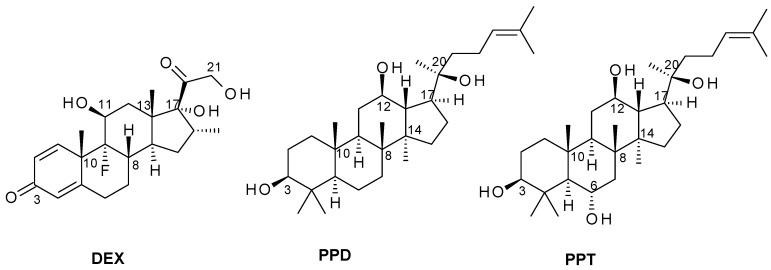
Chemical structures of the synthetic glucocorticoid DEX, a GR agonist, and the triterpenoids (20S)-Protopanaxadiol (PPD) and (20S)-Protopanaxatriol (PPT) studied in this work.

Triterpenoids, that share structural similarities with GCs proved to be potent and selective glucocorticoid receptor modulators. Experimental observations showed that triterpenoids, such as avidin D [25], echynocystic acid and its 3-O-glucoside derivative [26], induced GR nuclear translocation and GR transrepressional activities with no or limited GR transactivation. Some reports also demonstrate the ability of protopanaxadiol (PPD) and protopanaxatriol (PPT) to bind to nuclear receptors, such as estrogen and glucocorticoid receptor, and to control their actions [27,28]. Protopanaxadiol and protopanaxatriol share a dammarane-type tetracyclic terpene and belongs to the family of saponin triterpenoids, known as ginsenosides. Ginsenosides can be classified as protopanaxadiol-type or protopanaxatriol-type compounds which differ in the position, sugar substituents, and number of glycosyl attachments to the dammarane steroid backbone. PPD and PPT correspond to the aglycone form of the compounds. Furthermore, the C-20 of protopanaxadiol and protopanaxatriol is divided into 20(*S*) and 20(*R*)-type structures, depending on the position of the chiral carbon substitution [29] (Figure 1). Over 30 ginsenosides have been identified and classified into these two categories, among them the Rb1, Rb2, Rb3, Rc, Rd, Rg3, Rh2, Rs1 (the 20(*S*)-type protopanaxadiol) and the Re, Rf, Rg1, Rg2, Rh1 (the 20(*S*)-type protopanaxadiol [30]. The ability of ginsenosides to regulate the non-genomic effect of GR is also proposed [27]. Nevertheless, there is a controversy on whether PPD and PPT can regulate the genomic actions of glucocorticoid receptor [28]. Interestingly, PPD- and PPT- type ginsenosides are widely used for medicinal purposes, due to their pharmaceuticals activities, some of them similar to that of the glucocorticoids hormones. Thus, increasing numbers of studies reveal their potent anti-inflammatory, antidiabetic, cardioprotective, and apoptotic activities (reviewed by [29,31]). Nevertheless, the molecular and biochemical mechanisms of their pharmaceutical actions are unclear and not defined.

In this study the efficacy of the PPD and PPT compound to exert dissociative glucocorticoid like activity was examined in human HeLa, HEK293 and HepG2 cells, via assessment on their effect on GR potential binding, nuclear translocation, regulation of GR transactivation and transrepression activities and induction of apoptosis.

## 2. Results

### 2.1. Induced-Fit Docking Analysis Reveals Potential Binding of PPD and PPT to GR Ligand Binding Domain

Structural similarities of PPD and PPT with the natural cortisol and the synthetic corticosteroid, dexamethasone (DEX) (Figure 1), prompted us to examine the potential binding of PPD and PPT to the GR ligand binding domain (LBD), applying induced-fit docking analysis (IFD). For comparative purposes, therefore, DEX was also considered in these calculations. There are numerous examples of IFD used to successfully predict ligand-induced protein conformational changes and the effects on biochemical pathways in agreement with experiment [26,32,33,34,35], including for GR [26]. The results of the IFD are shown in Table 1, with both PPD and PPT predicted to bind well. The GlideScores are strong at ~ −15 and close to those of DEX (−15.59), while the IFDScores (−557.7 and −560.8 for PPD and PPT, respectively), that additionally take the protein energy/conformation into account, are in fact predicted better than for DEX (−551.7).

**Figure 2:**
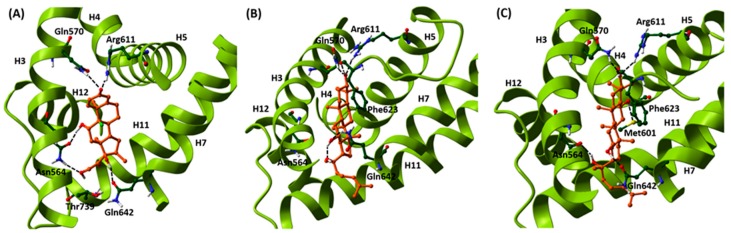
Binding of (**A**) DEX to the GR LBD from its crystal structure complex PDB code 1M2Z, together with the predicted binding of (**B**) PPD and (**C**) PPT from induced-fit docking calculation. Key protein residues are explicitly shown, while the ligands are displayed in orange.

The predicted binding interactions of PPD and PPT are shown in Figure 2. Both ligands occupy close to the same space as DEX, although the rings are not co-planar with DEX. In comparison, the RMSD between ring atoms of PPD and PPT is just 0.609 Å (aligned by superimposition of helices H5 and H8 backbone atoms [36]). The C-3 hydroxyl groups of both ligands exploit similar interactions to that of the DEX C-3 ketone group forming hydrogen bonds with Gln570 (H3) and Arg611 (H5). They are predicted to form an additional hydrogen bond with the Phe623 backbone O (β-strand between H5 and H6). The C-12 hydroxyls for both PPD and PPT are involved in important hydrogen bond interactions with the Gln642 (H7) sidechain. For PPT, the hydrogen bond is with the sidechain amide C=O similar to that formed by DEX through its C-17 hydroxyl. However, for PPD there is a flip in the Gln642 sidechain so that the hydrogen bond is instead between the sidechain NH_2_ and the C-12 hydroxyl O atom. This frees the C-12 hydroxyl H atom to form an intra-molecular hydrogen bond with the C-20 hydroxyl but it also closes to hydrogen bonding distance with the Asn564 (H3) sidechain carbonyl (an interaction present in the GR-DEX complex through its C-11 hydroxyl). For PPT, this hydrogen bond interaction with the Asn564 amide C=O is present through its C-20 hydroxyl group. The C-20 hydroxyl group of PPD, however, is not involved in any hydrogen bond interactions although it is close to, but not within hydrogen bonding distance of the previously mentioned flipped sidechain C=O of Gln642 (H7). Lastly, the C-6 hydroxyl of PPT, absent for PPD, forms weak hydrogen bond contact with the Met601 (H5) sidechain S atom. The tail group –CH_2_CH_2_CHC(CH_3_)_2_ of both PPD and PPT ligands is orientated between H7 and H11, and with no bad contacts.

Overall, therefore, although the hydrogen bond networks formed by PPD and PPT hydroxyls have differences, interactions with key residues for translation are observed similar to DEX, retaining helices such as H3 in it canonical position and exploiting other favorable hydrogen bond interactions with key residues in H5 (Arg611) and H7 (Gln642) [36]. A thorough analysis of GR interactions for steroidal and non-steroidal ligands from docking and solved crystallographic complexes in terms of triggering GR nuclear migration has previously been reported [36] and considering the antagonist RU486 (mifepristone), which also triggers nuclear migration, the evidence suggests that retaining H3 and H4 positions is more critical for translocation than the helical position of H12 (considerably shifted for RU486).

### 2.2. PPD and PPT Induce GR Nuclear Translocation

**Figure 3:**
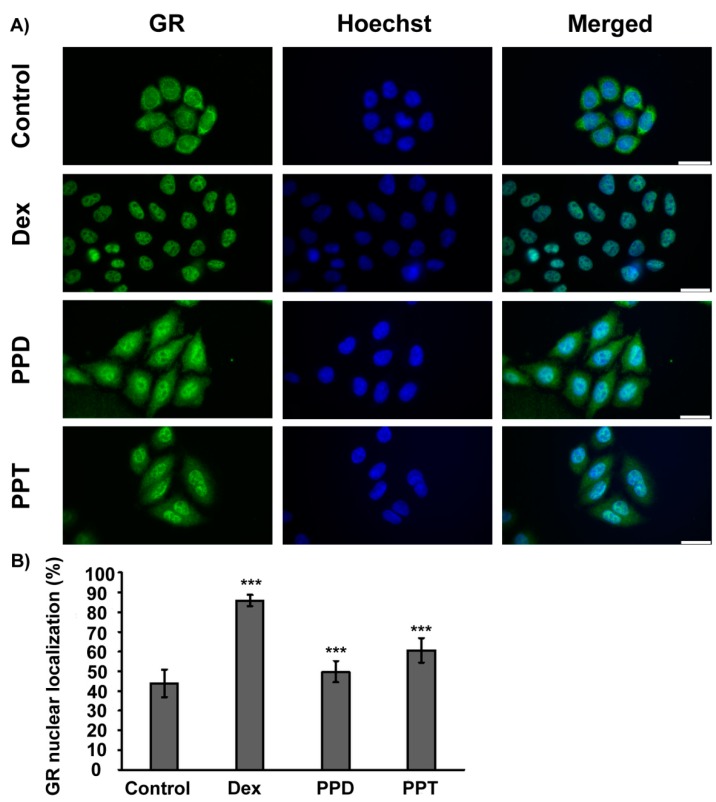
Induction of GR nuclear translocation by PPD and PPT. (**A**) HeLa cells cultured in hormone-free medium for 48 hrs were subsequently treated, with 10 μM of PPD or PPT, or 1 μM DEX, for 2 hrs. Control cells were treated with dimethyl sulfoxide (DMSO) (diluent of PPD and PPT) and EtOH (diluent of DEX), at the same dilution as steroid treated cells. Cells were then washed in phosphate-buffered saline (PBS), fixed in methanol-acetone and subjected to immunochemistry analysis using antibodies against GR (GR-H300), anti-rabbit secondary antibodies Alexa Fluor^®^ 488 conjugated (Green) and the Hoechst 33,342 (Blue) dye for nuclear staining. Bars indicate 30 μΜ. (**B**) Relative GR nuclear localization is expressed as percentage of the total corrected fluorescence of GR nuclear staining per the total corrected fluorescence of total GR cellular staining, assessed as described in experimental section. Data are expressed as mean ± S.D. (*n* > 20), *** *p* < 0.001, compared to control.

**Figure 4:**
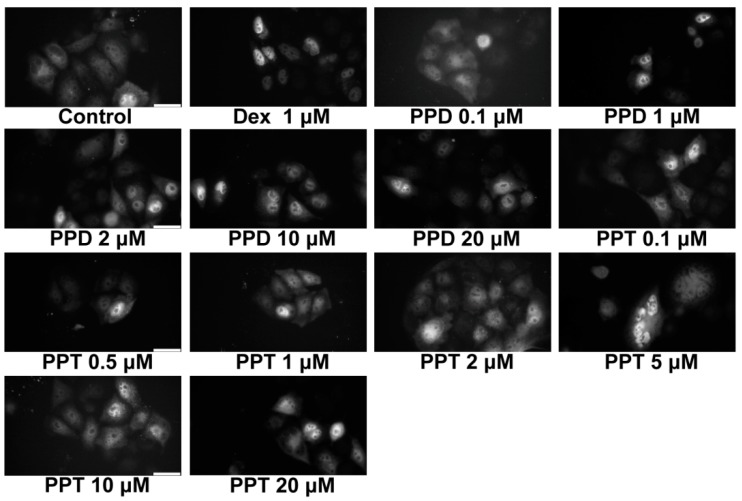
Dose-dependent PPD- and PPT- induced nuclear localization of GR. HeLa cells grown on coverslips, in hormone-free DMEM for 72 hrs, were subsequently transiently transfected with a pEGFPC2GR construct [8] and the next day cells were further incubated for 2 hrs with various concentrations of PPD or PPT, range from 0.1 μM to 20 μM, or 1 μM DEX. Cells were then washed with PBS, fixed in methanol acetone mounted in polyvinyl alcohol-based antifading medium and proceeded to microscopy analysis. Representative images of fluorescence microscopy analysis of GFPGR localization in HeLa cells, treated with various concentrations of PPD and PPT are presented. Bars indicate 30 μΜ. Quantification of the results is presented in Supplementary Figure S1.

Structural similarities of PPD and PPT with corticosteroids as well as results from the induced-fit docking analysis (Table 1), showing potential binding of PPD and PPT to GR, prompted us to explore the efficacy of PPD and PPT to induce GR nuclear translocation at cellular level. As shown in Figure 2, immunocytochemistry analysis of GR in HeLa cells, cultured in hormone-free medium, and treated with DEX, PPD or PPT, and statistical analysis of the results by one way ANOVA followed by Tukeys’ post-hoc test showed statistically significant increase in GR nuclear localization by DEX, PPD and PPT compound (F = 143.3; F_crit_ = 2.7; DF_between groups_ = 3; DF_within groups_ = 106; DF_total_ = 109, *p* < 0.001). 10 μM of PPD and PPT caused approximately 50 and 60 %, respectively, nuclear localization of GR, compared to the 85 and 40 % nuclear GR localization of the DEX-induced and vehicle-treated HeLa cells, respectively (Figure 3). In order to assess the dose dependency of this effect, HeLa cells, grown in hormone-free medium, were transiently transfected with the pEGFPC2GR construct [8] and subsequently treated with PPD or PPT compound, at concentrations range from 0.1 μΜ to 20 μΜ. Fluorescence microscopy analysis of the specimens revealed that concentrations higher than 0.1 μΜ are capable of inducing GR nuclear translocation (Figure 4). Quantification of the results showed a tendency of increase in GR nuclear translocation by the increased concentration of the PPT compound (Supplementary Figure S1). Statistical analysis of the results with one-way ANOVA followed by Fisher LSD test (but not with the Post Tukeys’ post-hoc test) showed statistical significant increase in GR nuclear translocation in group of cells treated with various concentrations of the PPT compound (F = 40.8; F_crit_ = 1.8; DF_between groups_ = 13; DF_within groups_ = 153; DF _total_ = 166, * *p* < 0.001).

### 2.3. PPD and PPT Suppress the DEX Induced GR Transactivation

**Figure 5:**
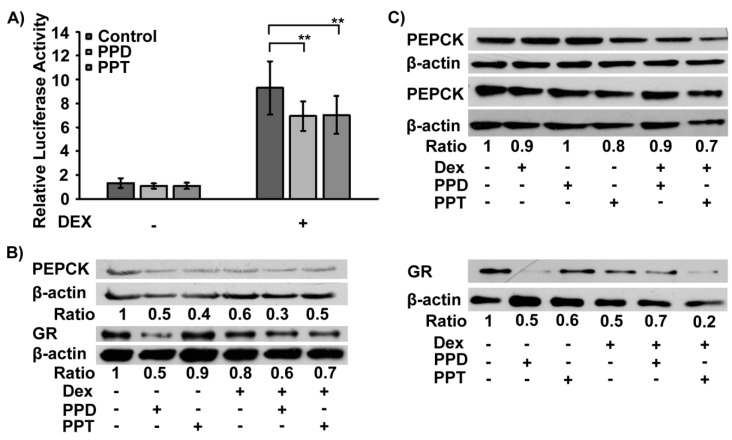
PPD and PPT did not induce GR transcriptional activation in HeLa cells. (**A**) Luciferase and β-galactosidase activity was measured in cell extracts from HeLa cells, cultured in hormone-free medium, transiently co-transfected with a GR-Luc reporter gene construct and a β-galactosidase reporter construct and subsequently treated with 10 μΜ of PPD or PPT and/or 1 μM DEX, for 6 hrs. Control cells were treated with DMSO (1:1000) and EtOH (1:1000). Relative luciferase activity was expressed as normalized luciferase activity relative to β-galactosidase activity. Results are expressed as mean ± S.D. ** *p* < 0.01. (**B**,**C**) Western blot analysis of PEPCK, GR, and β-actin protein levels was performed in extracts from HeLa (**B**) and HepG2 (**C**) cells treated with 10 μM PPD, 10 μΜ PPT and/or 1 μM DEX in hormone depleted medium, for 48 hrs. Commercially provided antibodies were used. Ratios indicate mean of ratios resulting from normalization of PEPCK or GR protein levels against β-actin from two independent experiments (Figure 5C, Supplementary Figure S3) or one (Figure 5B) experiment.

PPD- and PPT- induced GR nuclear translocation prompted us to evaluate the effect of both compounds on GR transcriptional activation. Luciferase reporter gene assays, using a MMTV-GRE (Mouse Mammary Tumor Virus-Glucocorticoid Response Elements) promoter-driven luciferase construct [37], revealed that the PPT and PPD-induced GR nuclear translocation was not accompanied by GR transcriptional activation (Figure 5A). As was expected, incubation of HeLa cells with 1 μΜ DEX caused approximately 4-5 fold statistically significant increase in GR transcriptional activity (F = 135.3; F_crit_ = 4.0; DF_within groups_ = 75; DF_total_ = 80, *n* > 10, *p* < 0.001, analysis of the results by two way ANOVA followed by Tukeys’ post-hoc test). Interestingly, PPT and PPD showed a suppressive effect against the DEX-induced GR transactivation. Incubation of the cells with DEX when combined with PPD and PPT showed approximately 20% inhibition of the DEX-induced GR transactivation. (F = 5.6; F_crit_ = 3.1; DF_within groups_ = 75; DF_total_ = 80, *n* > 10, *p* < 0.01). In addition, evaluation of the effect of the PPD and PPT compound on the GR target, gluconeogenic phosphoenolopyruvate carboxykinase (PEPCK) enzyme [38] showed decrease in PEPCK expression, in the presence or absence of DEX in HeLa cells, cultured in hormone-free medium (Figure 5B). Likewise, similar analysis in hepatocarcinoma HepG2 cells showed that treatment of the cells with PPT caused approximately 20–30% reduction in PEPCK protein levels, compared to control vehicle-treated cells (Figure 5C, Supplementary Figure S3). This effect may be associated with the observed reduction in GR protein levels in HeLa and HepG2 cells treated either with DEX or the PPD and PPT compounds, rather than their possible antagonistic effect on DEX transactivation (Figure 5C, Supplementary Figure S3).

### 2.4. Transcriptional Inactivation of NF-κΒ by PPD and PPT via Potential Activation of GR Transrepression Activity

**Figure 6:**
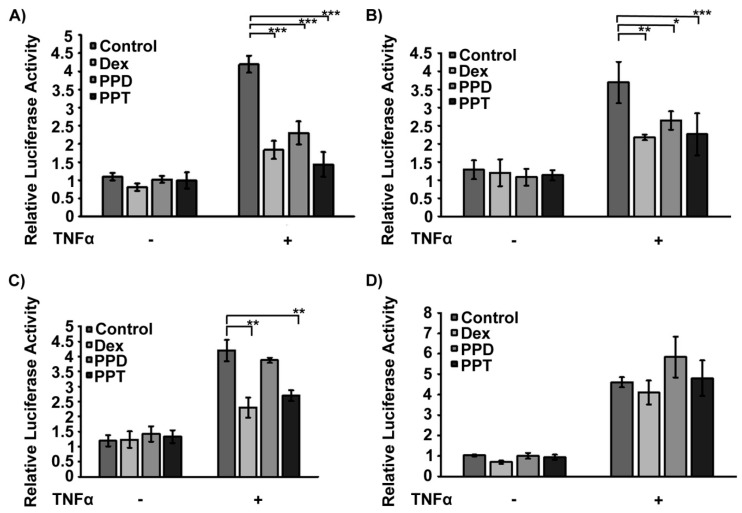
Suppression of the NF-κB transcriptional activity by PPD and PPT in HeLa and HEK293 cells possibly through regulation of GR signalling. (**A**,**B**) Luciferase and β-galactosidase activity was measured in cell extracts from GR positive HeLa cells cultured in hormone depleted medium, transiently co-transfected with a NF-κΒ Luc reporter gene construct and a β-galactosidase reporter construct and subsequently treated with 1 μM (**A**) or 10 μΜ (**B**) of PPD or PPT and/or 10 nM TNF α, for 6 hrs. Relative luciferase activity was expressed as normalized luciferase activity against β-galactosidase activity. Effect of 10 μΜ of PPD or PPT on NF-κB activity was also evaluated in GR positive HEK293 cells (**C**) and in GR low level COS-7 cells (**D**). Data are expressed as mean ± S.D. (*n* = 3–5), * *p* < 0.05; ** *p* < 0.01; *** *p* < 0.001.

Anti-inflammatory activity of PPD and PPT was assessed via evaluation of their actions on NF-κB activity. Thus, effect of PPD and PPT on the NF-κΒ activity was assessed in GR-positive HeLa (Figure 6A,B) and HEK293 (Figure 6C) cells, as well as in GR low level COS-7 cells (Figure 6D), grown in hormone-free medium, and transiently transfected with a NF-κB-Luciferase (NF-κB-Luc) and a β-galactosidase construct [37]. HeLa (Figure 6A,B) and HEK293 (Figure 6C) were subsequently treated with Tumor Necrosis Factor α (TNF α) (10 ng/mL) or equal volume of PBS, in the presence or absence of 1 μΜ or 10 μΜ of PPD or PPT, 1 μΜ DEX or DMSO/EtOH vehicle (Figure 6A,B). Measurements of luciferase activity revealed that TNF α induced statistically significant increase in NF-κB transcriptional activation, in both cell lines ((A) F = 214.4; F_crit_ = 4.5; (B) F = 78.1; F_crit_ = 3.9; (C) F=276.4; F_crit_ = 4.5, *p* < 0.001), whereas DEX caused statistically significant suppression of the TNF α - induced transcriptional activity of NF-κΒ, by 40–55 %. A similar effect was also observed by PPD and PPT ((A) F = 35.2; F_crit_ = 3.2; DF_within groups_ = 16, DF_total_ =23; (B) F = 10.9; F_crit_ = 2.7; DF_within groups_ = 92; DF_total_ = 99; (C) F = 4.4, F_crit_ = 3.2; DF_within groups_ = 16; DF_total_ = 23). PPD suppressed the TNF α-induced NF-κΒ activity by 28–45%, whereas PPT exerted 38–65 % inhibition. Our results indicate that increase in PPD and PPT concentration from 1 μΜ to 10 μM did not substantially strengthen their inhibitory activity. Evaluation of the anti-inflammatory effect of 10 μM of PPD and PPT, via transcriptional inactivation of the TNF α-activated NF-κΒ, was not observed in GR low level COS-7 cells (Figure 6D) (F = 87.4, F_crit_ = 4.5, (−TNF/+TNF); F = 0.8, F_crit_ = 3,3 (Treatment); F = 2.0, F_crit_ = 3.2, (TNF/Treatment); DF_within groups_ = 16, DF_total_ = 23); “Treatment” corresponds to treatment either with vehicle, DEX, PPD or PPT), providing circumstantial evidence for the involvement of GR in the PPD- and PPT-induced NF-κΒ transcriptional inactivation. Cytotoxicity analysis in HeLa cells, applying the sulphorodamine B assay [39], showed that 48 h incubation of HeLa cells with the PPD and PPT compounds, at a concentration range from 1 μΜ to 20 μΜ, caused no statistical significance inhibition in cell growth. This effect indicates that the observed anti-inflammatory activity of the compounds is not due to any cytotoxic effect (Supplementary data, Supplementary Figure S2).

### 2.5. PPD and PPT Induce Mitochondrial-Dependent Apoptosis

**Figure 7:**
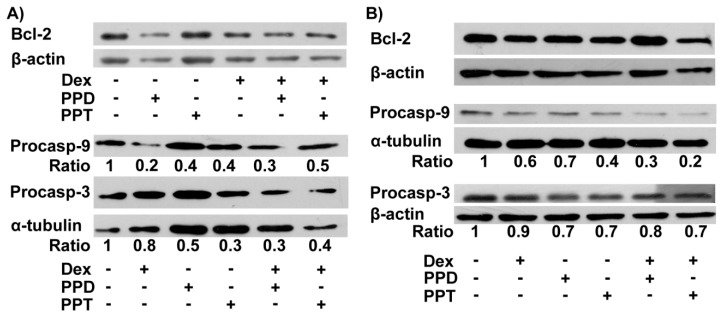
PPD and PPT induce mitochondrial dependent apoptosis. Western blot analysis of β-actin, Bcl2, pro-caspase 3 and pro-caspase 9 protein levels in cell extracts from HeLa (**A**) and HepG2 (**B**) cells treated with 10 μM PPD or PPT and/or 1 μM DEX, for 6 hrs in hormone depleted medium, was performed using commercially provided antibodies. Ratios indicate normalization of Bcl2, pro-caspase 3 and pro-caspase 9 protein levels against β-actin or α-tubulin, as indicated.

In order to evaluate the effects of PPD and PPT on apoptosis, Western blot analysis of anti-apoptotic and apoptotic molecules such as Bcl2, procaspase 3 and procaspase 9 was performed in HeLa (Figure 7A) and HepG2 (Figure 7B) cells, treated or not with 1μΜ DEX and/or 10 μΜ of the PPD and PPT compounds, for 48 hrs. As shown in Figure 7, evaluation of the effect of the PPD and PPT compound on Bcl2 protein level in HeLa and HepG2 cells, showed no remarkable action. Interestingly, as shown in Figure 7A, DEX, PPD, and PPT compounds caused induction of the mitochondrial dependent apoptosis, in HeLa cells, as indicated by the observed reduced protein level of the uncleaved, non-activated procaspase 9 and procaspase 3, compared to control, vehicle treated HeLa cells (Figure 7A). The mitochondrial dependent induction of apoptosis by PPT and PPD was also verified in HepG2 cells, as indicated by the decreased protein level of the uncleaved caspase 9 and caspase 3, upon treatment of the cells with 10 μM of the PPD and PPT compounds. In addition, a moderate increase in caspase 9 activation is observed upon co-administration of DEX with the PPD and PPT compounds (Figure 7B).

## 3. Discussion

Triterpenoids are structurally diverse organic compounds that exist widely as natural products. These compounds are precursors of steroids in both plants and animals [40]. Triterpenoid saponins, known as ginsenosides, are biologically active chemical in ginseng, a traditional herbal adaptogen, of the genus Panax (Araliance Family) that has been widely used in traditional medicine because of its medical properties [31]. Ginsenosides biological activities include antidiabetic actions, hepatoprotective activity, anti-inflammatory effect, myocardial protection, lipid regulation, antioxidation, neuroprotection, anti-angiopathy and anti-neurotoxic effects [29,31].

Pharmacological activities of ginsenosides are mainly attributed to their steroidal structure, which enables them to diffuse across cellular membrane, to enter the cells and to interact with membranous, cytoplasmic and nuclear target proteins, modulating their activities and cellular functions. Thus, experimental data indicate that ginsenosides interferes with signaling pathways such as phosphotidyloinositol-4,5 bisphosphate 3-kinase/protein kinase B (PtdIns(4,5)P2/Akt) pathway, signal transducer and activator of transcription 5/peroxisome proliferator-activated receptor gamma coactivator 1-alpha (STAT5/PPARgamma) pathway, phosphoinositide-3 kinase/protein kinase B (PI3K/Akt) pathway, AMP-activated protein kinase/c-Jun terminal NH2-terminal kinase (AMPK-JNK) pathway, NF-κB pathway, endoplasmic reticulum stress, inflammatory, antioxidant and apoptotic pathways, glucose import and glucose metabolism pathways [29]. Nevertheless, the biochemical and potential pharmacological mechanisms, as well as the molecular targets of ginsenosides are unclear and remains to be elucidated.

Due to the structural similarities of ginsenosides with steroid hormones they could be considered as potential modulators of steroid hormone receptors. Accumulating evidence indicate that ginsenosides are involved in the regulation of nuclear receptors [41]. Thus, it has been shown that PPD- and PPT- type ginsenosides are capable of binding to GR [42,43,44,45,46]. The efficacy of binding is lower to that of the synthetic glucocorticoid DEX, and more importantly, experimental data, resulting from a comparative time-resolved fluorescence resonance energy transfer competitive ligand binding assay, showed that the number of glycosylated groups in ginsenosides is accompanied by a decrease in receptor binding potency [28]. In addition, accumulating studies showed that ginsenosides metabolites had greater biological effects than ginsenosides [30]. As regards the effect of certain PPT- and PPD- type ginsenosides on GR transactivation activity and transrepression activity, conflicting data are presented [28,43,46,47,48,49,50,51,52]. Nevertheless, to our knowledge, there is a lack of data on the effect of the aglycone form of PPT- and PPD- type compounds on GR transactivation and transrepression activities.

Our data from induced fit docking analysis showed that the aglycone form of the PPD- and PPT- type compound is capable of binding to GR ligand binding domain. In fact, comparable binding strengths to DEX are predicted as well as similar interactions with GR LBD helices/residues, previously cited as important for nuclear translocation [26,36]. As we have previously stated [26], the crucial factor is the ligand-dependent structural reorganizations of the LBD that allow a receptor to function as a more potent activator. PPT is predicted to bind slightly stronger than PPD in the predicted docking models (Table 1). The potential efficacy of PPD and PPT to bind to GR ligand binding domain is in agreement with the results from the comparative study of [28] and is further confirmed by our data showing that PPD- and PPT- induced GR nuclear translocation at cellular lever, in HeLa cells. The observed more favorable PPT- translocation is also consistent with predicted stronger interactions with H3 through direct hydrogen bonding interaction with the Asn564 amide C=O. PPD and more effectively the PPT compound are capable of inducing endogenous as well as gene delivered-induced GR nuclear translocation at concentrations ranging from 0.1 μΜ to 20 μΜ.

PPD- and PPT- induced nuclear translocation was not accompanied by GR transactivation, as indicated by the results from the GRE-driven luciferase reporter gene expression assay, showing no PPD- and PPT- induced GRE driven luciferase gene expression. Nevertheless, both compounds were capable of suppressing the DEX-induced GR transactivation. Moreover, PEPCK protein level were considerably decreased in HeLa cells, cultured in hormone-free medium, and treated with PPT or PPD in combination or not with DEX. In addition, moderate reduction in PEPCK protein levels by PPT, but not by PPD, was observed in HepG2 cells. Since liver is the main gluconeogenic organ, the discrepancy of the results in the two cell lines may be attributed to the more pronounced gluconeogenetic properties, of the hepatocarcinoma HepG2 compared to that of the HeLa cells, relied on the differential expression of GR and its co-activators or co-repressors, as well as, to the differential recruitment of these factors in the GR transcription complex, triggered by binding of PPD or PPT to GR [53]. Our observation of the reduction of PEPCK protein level by PPD and PPT is consistent with previous findings demonstrating that certain ginsenosides such as Rb1 (multiglycosylated PPD-type ginsenoside) induced reduction in hepatic glucose production by suppressing the expression of the gluconeogenic glucose 6-phosphate phosphatase and phosphoenolpyruvate carboxykinase enzymes [54]. The reduction in PEPCK protein levels in PPD- and PPT- treated cells could be associated with the reduction in GR protein levels, observed in HepG2 cells, upon treatment with the PPD and PPT compound. Reduction in GR protein levels by the PPD compound was also verified in HeLa cells. Reduction in GR protein levels by PPT and PPD may be the result of the activation of AMP-activated protein kinase, (AMPK). Activation of the AMPK is demonstrated to take place upon conditions of glucocorticoids-stress [55], but also upon treatment with the Rb1 ginsenoside [54]. In addition, in line with our observation, and in support of the notion of a potent GCs-like activity of the compounds, reduction in GR protein level, in the presence of DEX at concentrations higher than 10^−7^ M, has also been reported in various types of cells, including HeLa cells [26,56,57,58]. The ligand–induced repression of the GR gene is proposed to be mediated by an NCoR1 (nuclear repressor co-repressor 1) repression complex formation [59], however, GCs non-genomic mechanisms of action could also be involved in this action. Thus, although DEX induced GR transcriptional activation, this effect is compensated by the DEX-induced reduction in GR protein levels, resulting in not profound increase in PEPCK protein levels compared to control cells.

The antidiabetic-hypoglycemic and anti-inflammatory activity of PPD- and PPT- type ginsenosides has been known for a very long time. The molecular mechanisms underlying these actions are unclear and under investigation. Thus, multiglycosylated PPD- and PPT-type ginsenosides, such as Rb1 and Rg1, respectively, have been shown to exert antihyperglycemic activity via induction of increase in insulin sensitivity and insulin uptake [60,61], suppression of insulin resistance [62], reduction in food uptake and body weight [54], increase in membrane translocation of glucose transporters GLUT1 and GLUT4 [61,63], increase in glucose uptake [54] and suppression of the gluconeogenic enzymes glucose 6-phosphate and PEPCK expression [54,64].

Even though PPD and PPT did not induce GR transactivation, both compounds were capable of suppressing the TNF-α -induced NF-κB activity in HeLa- as well as in HEK293- GR positive cells. 1 μM of the compounds were approximately as effective as 10 μM to suppress the TNF-α -induced NF-κB activity in HeLa cells, in agreement with results from fluorescence microscopy analysis, showing that concentrations higher than 1 μΜ of the compounds are effective enough to trigger the GR nuclear translocation. Similar effects have also been observed by the synthetic glucocorticoid, DEX. Thus, the ability of DEX to fully activate GR nuclear translocation and transcriptional activation, in a broad range of concentrations, varying from nM to μΜ, has been reported [26,65]. Furthermore, the PPT compound, that is more active in activation of the GR nuclear translocation, is also more active in suppression of the NF-κB activity. Interestingly, PPT is as effective as DEX, causing approximately 40% reduction in the TNF-α induced NF-κΒ activity. The suppressive effect of the compounds in not attributed to any inhibitory effect of the compounds on cell growth, as indicated by results from the sulphorodamine B assay. Moreover, the inhibitory effect of PPD and PPT on the NF-κΒ activity is abolished in GR low level COS-7 cells, providing circumstantial evidence for the involvement of GR in this process.

The suppressive effect of PPD and PPT on NF-κΒ activity in combination with their inability to induce GR transactivation renders them potential promising lead molecules for the development of novel potent drugs with anti-inflammatory activity but with no or reduced side effects. Moreover, the possible additive effect of the compound when administered in combination with other GR modulators, that also exert GR transrepression activity, may be of significant pharmacological importance. Thus, co-administration of the PPD and PPT compounds with RU486, the well-known GR antagonist which also exert immunosuppressive actions, through suppression of NF-κB, AP1 activity and cytokines production [66,67,68], could possibly lead to further enhancement of the PPD and PPT anti-inflammatory activity. Nevertheless, due to a possible competition between PPD/PPT and RU486 for binding to GR, none or just a partial reversal of the suppressive PPD or PPT effect is also likely to occur. In addition, taking into account the importance of the differential conformational GR changes induced upon binding, as well as other factors, such as type of the cell, presence or absence of corepressors/coactivators or other regulatory molecules, that affect the final outcome of the co-administration of the compound [20], a cell type-specific action could be arise with potential pharmacological importance.

In line with our observations, multiglycosylated PPD- and PPT-type ginsenosides such as Rb1 and Rg1 have been shown to exert anti-inflammatory activity, through (a) reduction in pro-inflammatory cytokines and mediators, including TNF-α, interleukins IL-6, IL-1β, (b) regulation of ΝF-κB and JNK signaling pathway, and (c) increase in nitric oxide synthesis and release [69,70,71]. Glucocorticoids signaling also involve interference with these pathways [72,73], thus binding of PPD and PPT to glucocorticoid receptor and regulation of its action may be at least in part the causative for these effects.

Glucocorticoids are also well known for their tissue and cell type-specific pro-apoptotic actions [74] rendering them effective and widely used drug for the treatment of certain lyphoproliferative disorders and to alleviate side effects induced by chemotherapy or radiotherapy in nonhematologic cancer types [14].

In this study we showed that PPD and PPT compound exert apoptotic activities, which are probably mitochondrial-mediated as indicated by the activation of caspase 9 and 3 upon PPT and PPD incubation of HeLa and HepG2 cells. In addition, co-incubation of HepG2 cells with DEX and the PPD or PPT compound caused increase in procaspase 9 indicating, cell type-specific interference of PPD and PPT compounds with GR signaling. The observed induction of apoptosis is indicated to be at early stage, since reduction in procaspase 9 protein level is higher than that of the pro-caspase 3, in the case of HepG2 cells, and also, no statistical significance inhibition of HeLa cells growth by the PPD and PPT compound was observed, at this time point. The exact mechanism of induction of apoptosis remains to be elucidated. However, it is possibly related to the steroid-like activities of the compounds.

To conclude, in this study, we provide evidence that glucocorticoid receptor may constitute, among others, a potential molecular target for PPD and PPT compounds, to drive their pharmaceutical actions. Thus, we showed that PPD and PPT compound can effectively bind to GR and activate its nuclear translocation. Activated GR exert transrepression activity, suppressing NF-κB activation, whereas no transactivation of GR was observed. Moreover, reduction in PEPCK expression by PPD and PPT was observed, possibly due to the induced reduction in GR protein levels. To our knowledge this is the first study to describe potent anti-hyperglycemic, anti-inflammatory actions and apoptotic activities of the aglycone form of the PPD- and PPT-type ginsenoside, revealing also potential dissociative activity of PPD and PPT compound on GR transcriptional activation. Although further analysis is required to delineate the exact mechanisms of actions and interfering of PPD and PPT with GR signaling, our study contribute to the establishment of new lead molecules for the development of selective glucocorticoid receptor agonists with potential for therapeutic use as anti-inflammatory and anticancer drug, with reduced side effects.

## 4. Materials and Methods

### 4.1. Chemicals

Dulbecco’s modified Eagle’s medium (DMEM), fetal bovine serum (FBS), and lipofectamin 2000 were obtained from Invitrogen (Life technologies corporation, Grand island, NY, USA). Molecular weight protein markers were from Fermentas (Thermo Fisher Scientific GmbH, Frankfurt, Germany) complete protease inhibitors cocktail were purchased from Roche (Mannheim, Germany) and TNFα was purchased from Immuno Tools (Friesoythe, Germany) All other chemicals including dexamethasone (DEX) were purchased from Sigma-Aldrich (St. Louis, MO, USA). PPD and PPT were purchased from Extrasynthese, Genay Cedex, France.

### 4.2. Antibodies

The GR-H300 affinity purified polyclonal GR antibody, which recognizes an epitope corresponding to amino acids 121-420 of human GR, and was commercially provided by Santa Cruz Biotechnology, was used. Monoclonal antibodies against β-actin (Sigma Aldrich, St. Louis, MO, USA) and caspase 9 (Cell Signalling Technology, Leiden, The Netherlands), and rabbit polyclonal antibodies against phosphoenolpyruvate carboxykinase (PEPCK) (Santa Cruz Biotechnology, Inc, Europe, Heidelberg, Germany), caspase 3 (Abcam, Cambridge, United Kingdom), Bcl-2 (Cell Signalling Technology, Leiden, The Netherlands) were also applied.

### 4.3. Cell Lines-Cell Culture

The human hepatocarcinoma HepG2 and HEK293 cells were obtained from the American type culture collection (ATTC). HeLa and COS-7 cells were a kind gift from Dr. M Alexis, from the National Hellenic Research Foundation (NHRF) Greece. All cell lines were maintained in DMEM, supplemented with 10% FBS, 2 mM glutamine, and 100 Units/mL penicillin/streptomycin. Cells were cultivated at 37 °C in 5% CO_2_ humidity. 48–72 hrs before treatment, cells were cultured in phenol red free-DMEM medium supplemented with 10% charcoal-dextran- stripped FBS (charcoal inactivated FBS), 2 mM glutamine, and 100 Units/mL penicillin/streptomycin.

### 4.4. Immunofluorescence Microscopy

Cells grown on coverslips, in DMEM hormone-free medium (DMEM without phenol red supplemented with 10% charcoal inactivated serum) for 48 hrs. Cells were then transiently transfected with a pEGFPC2GR construct [8]. After 24 hrs, cells were subjected to 2 hrs treatment with PPD or PPT at concentrations range from 0.2 μM to 20 μM, or 1μM DEX. Cells were then washed with PBS, fixed for 10 min at −20 °C in methanol, transferred to acetone (−20 °C) for 2 min, briefly air-dried and mounted in polyvinyl alcohol-based anti-fading medium. In immunocytochemistry experiments, cells grown on coverslips, in DMEM without phenol red supplemented with 10% charcoal inactivated serum, were incubated either with 10 μM PPD/PPT or 1 μM dexamethasone (DEX), for 1 hr, at 37 °C, washed with PBS, and fixed in methanol-acetone (−20 °C). After three washings (5 min each), immunocytochemistry was proceeded using primary GR antibodies (final dilution of 1:50), appropriate secondary antibodies conjugated with Alexa fluor 488, provided by Invitrogen (Life technologies corporation, Grand island, NY, USA), diluted 1/500, and 1 μM Hoechst 33,342 (Sigma-Aldrich, St. Louis, MO, USA). Specimens were mounted in polyvinyl alcohol-based anti-fading medium [8]. Cell specimens were observed with a Leica 2000 DM microscope (Leica, Heerbrugg, Switzerland). Images were obtained with the optiMOS camera (Qimaging, Surrey, BC, Canada) which kindly donated by the Bodossaki Foundation, Greece. Quantifications of the relative GR nuclear staining was performed by the use of the ImageJ v1.47 program (NIH, Bethesda, MD, USA) as previously described [75]. Briefly, in each individual cell two areas of interest were drawn. One based on total green fluorescence staining (total) and the other based on Hoechst staining (nuclear). Mean green fluorescence and integrated density was measured along with several adjacent background readings. The total corrected fluorescence of area of interest (TCF) = integrated density – (selected area × mean fluorescence of background readings), was calculated. Relative GR nuclear localization is expressed as percentage of the nuclear TCF of GR staining per the TCF of total GR cellular staining.

### 4.5. NF-κB and GR Activity

NF-κB and GR transcriptional activity was measured applying luciferase reporter gene assay. Briefly, HeLa cells grown on 24-well plates were co-transfected, using Lipofectamin 2000, with a NF-κB-Luciferase (NF-κB-Luc) (for assessment of NF-κB activity), or an MMTV-GRE (Glucocorticoid response elements of the murine mammary tumour virus DNA) promoter-driven luciferase construct (GR-Luc reporter gene construct) (for assessment of GR activity) and a β-galactosidase reporter construct [8,37]. The next day cells were triggered either by TNF α (tumour necrosis factor α; 10 ng/mL) for 6 hrs (for assessment of NF-κB activity), or by 1 μM DEX (for assessment of GR activity), in the presence or absence of the indicated amounts of PPD, PPT or DEX. Subsequently, cells were lysed in report lysis buffer (Promega Coorporation, Madison, USA), and the enzymatic activities of the expressed luciferase and β-galactosidase were measured [37]. The light emission was measured using a chemiluminometer (LB 9508, www.berthhold.com). Relative luciferase activity was expressed as normalized luciferase activity against β-galactosidase activity (RLU).

### 4.6. Electrophoresis and Western Blotting

Cells grown on 6 well plates, for 48 hrs in hormone depleted medium, were incubated for additional 48 hrs with 10 μΜ of PPD, 10 μΜ of PPT and/or 1 μΜ DEX. Cells were washed in PBSX1, lysed in buffer A (20 mM Tris pH:7.5, 250 mM NaCl, 0.5 % Triton, 3 mM EDTA) supplemented with cocktail preotease inhibitors (Rosche, Germany). After Bradford protein determination [76], cell extracts were electrophoresed in discontinuous SDS-PAGE and Western blotted with specific antibodies against GR, β-actin, α-tubulin, PEPCK, Bcl-2, caspase 3 and caspase 9 as previously described [77]. β-actin or α-tubulin expression levels were evaluated for the normalization of the PEPCK, GR, Bcl-2, procaspases-3 and -9 expression levels. Enhanced chemiluminescence was used for the detection of the protein bands.

### 4.7. Statistical Analysis

All results are expressed as mean ± SD. Data were analysed by independent *t*-test or by One-Way analysis of variance (ANOVA) (Figure 3) or Two-Way ANOVA (Figure 5 and Figure 6) followed by Tukeys’s post-hoc test using StatPlus software. Differences were considered significant at a two tailed *p* value < 0.05. Further details for statistics are given in the Supplementary Information, Section 4: Full statistics—Details on the statistical analysis of the results.

### 4.8. Induced-Fit Docking Calculations

The ligands (PPD, PPT and DEX) were prepared for docking calculations using LigPrep v3.6 [78] with the OPLS3 forcefield [79]. Initial 3D structures of the two triterpenoids were downloaded from PubChem (https://pubchem.ncbi.nlm.nih.gov/). The GR protein was prepared for docking using the solved crystallographic complex (PDB code: 3BQD) with deacylcortivazol, exploiting its more open binding pocket for the study of bulkier ligands [26,80], and previously successfully applied to investigate the binding properties of echinocystic acid and its 3-*O*-glucoside derivative [26].

The initial setup of the GR structure for calculations was performed using Schrodinger’s “Protein Preparation Wizard” [78]. Water molecules within 5 Å of the deacylcortivazol ligand were initially retained but deleted for subsequent docking. Bond orders were assigned, hydrogen atoms added, with protonation states for basic and acidic residues based on calculated PROPKA pK_a_ values at pH 7. Subsequent optimization of hydroxyl groups, histidine sidechain C/N atom “flips” and protonation states, and side-chain O/N atom flips of Asn and Gln was based on optimizing hydrogen bonding patterns. Finally, an “Impref” minimization was performed using the OPLS3 force field [79] to remove any steric clashes/bad contacts but with heavy atoms constrained to within 0.3 Å (RMSD) of their original positions.

IFD calculations [78] consisted of three stages. In Stage I, initial docking using Glide v7.2 in SP (standard precision) mode was performed with docking grids of dimensions 29 Å × 29 Å × 29 Å centred on the deacylcortivazol ligand, with a maximum of 20 poses saved. Stage II was a Prime v4.5 induced-fit using OPLS3 with protein residues within 7.5 Å of the initial ligand poses refined [26]. In the final Stage III, up to 20 structures from Stage II within 30 kcal/mol of the lowest energy structure were used for Glide-XP (extra-precision) ligand re-docking calculations. The final protein-ligand geometries were analyzed in terms of structure and binding interactions, ligand re-docking GlideScores (GSs), as well as IFDScores (re-docking GS + 5% Prime energy).

## Figures and Tables

**Table 1:** Induced-fit docking calculation results ^a^.

Ligand	GlideScore	IFDScore
DEX ^a^	−15.59	−551.7
PPD ^b^	−14.93	−557.7
PPT ^c^	−15.23	−560.8

^a^ Top-ranked pose based on IFDScore which had a ligand heavy atom RMSD (root-mean-square deviation) just 0.610 Å compared with its crystallographic conformation (PDB code: 1M2Z). The RMSD was calculated by overlapping the predicted complex against 1M2Z by matching the residue backbone atoms of helices H5 and H8 [36]. ^b^ Fourth ranked IFDScore pose: exhibited interactions in the LBD favorable for nuclear translocation [26,36]. For comparison, the top ranked PPD protein-ligand pose had a GlideScore and IFDScore of −13.89 and −558.6, respectively, and hence had less favorable interactions in the LBD based on GlideScore. ^c^ Top-ranked pose based on the IFDScore: exhibited binding characteristics consistent with nuclear translocation [26,36].

## Supplementary Materials

Supplementary materials can be found at http://www.mdpi.com/1422-0067/20/1/94/s1.

## Abbreviations

Akt	protein kinase B
AMPK	AMP activated protein kinase
ANOVA	Analysis of Variance
DEX	Dexamethasone
GCs	Glucocorticoids
GLUT	glucose transporters
GR	glucocorticoid receptor
GRE	Glucocorticoid Response Element
IFD	Induced-Fit Docking Analysis
IL	Interleukin
JNK	c-Jun terminal NH2-terminal kinase
NF-κΒ	Nuclear factor-kappa beta
LBD	Ligand Binding Domain
MMTV	Mouse Mammary Tumor Virus
PEPCK	Phosphoenolopyruvate Carboxykinase
PI3K	phosphoinositide-3 kinase
PPARgamma	peroxisome proliferator-activated receptor gamma coactivator 1-alpha
PPD	Protopanaxadiol
PPT	Protopanaxatriol
PtdIns(4,5)P2	Phosphotidyloinositol-4,5 bisphosphate 3-kinase
SEGRAs	Selective Glucocorticoid Receptors Activators
SEGRMs	Selective Glucocorticoid Receptors Modulators
Stat3	Signal Transducer and Activator of Transcription – 3
STAT5	signal transducer and activator of transcription 5
TNFα	Tumor Necrosis Factor α
