# Supplementary material for: Potential Dissociative Glucocorticoid Receptor Activity for Protopanaxadiol and Protopanaxatriol
Source: Int J Mol Sci. 2018 Dec 27;20(1):94. doi: 10.3390/ijms20010094 (PMC6337468; doi:10.3390/ijms20010094)
Supplement: Supplementary file 1 [file ijms-20-00094-s001.pdf]

Supplementary Data

Potential dissociative glucocorticoid receptor activity for protopanaxadiol and protopanaxatriol

Aikaterini G. Karra[1], Maria Konstantinou[1], Maria Tzortziou[1], Ioannis Tsialtas[1], Foteini D. Kalousi[1], Constantine Garagounis[1], Joseph M. Hayes[2], and Anna-Maria G. Psarra[1,*]

[1] Department of Biochemistry and Biotechnology, University of Thessaly, Larissa, Greece
[2] School of Pharmacy & Biomedical Sciences, University of Central Lancashire, Preston PR1 2HE, UK
* Correspondence: ampsarra@bio.uth.gr; Tel.: +30-2410-565221

1. *Quantification of the PPD- and PPT- induced GFPGR nuclear translocation in HeLa transfected cells*

Quantifications of the GR nuclear localization in GFPGR transfected HeLa cells treated with the PPD and PPT compounds at a concentration range from 0.1 μM to 20 μM was performed by the use of the ImageJ v1.47 program (NIH, Bethesda, MD, USA). Briefly, for the assessment of the relative nuclear staining in each individual cell two areas of interest were drawn. One based on total green fluorescence staining (total) and the other based on Hoechst staining (nuclear). Mean green fluorescence and integrated density was measured along with several adjacent background readings. The total corrected fluorescence of area of interest (TCF) = integrated density − (selected area × mean fluorescence of background readings), was calculated. Relative GR nuclear localization is expressed as percentage of the nuclear TCF of the GFPGR staining per the TFC of total GFPGR cellular staining.

Analysis of the results by independent t-test or by one way analysis of variance followed by Tukeys's post-hoc test using StatPlus software showed that 1 μM DEX, as well as PPD and PPT compounds at concentration range from 0.1 μM το 20 μM, exhibited statistically significant induction in GFPGR nuclear translocation compared to control vehicle treated cells (F=40.8; F_{crit}=1.8; $DF_{between\ groups}$=13; $DF_{within\ groups}$= 53; DF_{total} =166, *p<0.001). In addition, a tendency of a dose dependent increase in GR nuclear translocation, by the more effective, as regard repression of the NF-κB activity, PPT compound, was observed, which is considered statistically significant upon analysis by one way ANOVA followed by Fisher LSD test. The maximum activation of GR, at a broad range of concentration of PPD and PPT, resembles that of DEX at concentration range from 0.1 μM to 1 μM, as regards induction of nuclear translocation [26], as well as induction in GR transcriptional activation (concentration range of 10 nM to 1 μM) [64]. The difference in the magnitude of GR translocation in GFPGR overexpressing cells compared to that of the endogenous expressed GR in HeLa cells may indicate that the GFP-tagged GR experiment is likely to amplify the signal since this is an overexpression line, thus making the system more sensitive. Additionally, it limits background fluorescence, since GFP fluorescence is directly proportionally to the number of GFPGR molecules present in the cytosol and nucleus and not altered by potential artifacts of antibody-incubation, background staining and non-specific interactions of primary and secondary antibodies used in immunocytochemistry. Thus, studies on GR transfected cells may constitute a better approach to evaluate the efficiency of potential glucocorticoid receptor modulators in nuclear translocation induction.

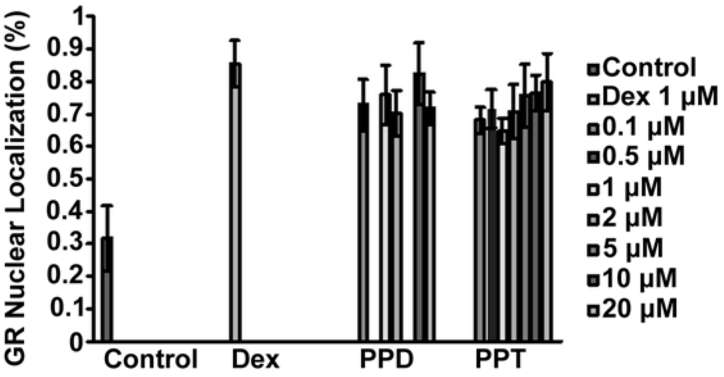

Figure S1. Quantification of the PPD- and PPT- induced nuclear translocation of the GFPGR in HeLa transfected cells. 1μM DEX and PPD and PPT compounds at concentration range from 0.1 μM to 20 μM exhibited statistically significant induction in GFPGR nuclear translocation compared to control vehicle treated cells. Results are expressed as means ± SD, n>10,**p*<0.001). No statistical significance was observed between groups of cells treated with various concentration of each compound, analysed by one way ANOVA followed by Tukeys' post-hoc test. However, statistical analysis of the results by one way ANOVA followed by Fisher LSD test showed statistical significant increase in nuclear GFPGR localization in cells treated with various concentrations of PPD and PPT as indicated below (Paragraph 4, Full statistics).

2. *Cytotoxicity assay- Effect of PPD and PPT on cell growth inhibition*

Cytotoxicity of the PPD and PPT compounds was assessed by applying the sulphorodamine B (SRB) assay according to Vichai & Kirtikara (2006)[39] with some modifications. Briefly, HeLa cells were seeded in 96 plates at a density of 1.1 X 10^4 cells per well. After 24 hrs cells, in wells in triplicates, were treated with increasing concentrations of PPD and PPT (1 μM – 20 μM) or 1 μM DEX, in DMEM growth medium supplemented with 2 mM glutamine, and 5% FBS, as indicated in supplementary Figure S2. Control cells were treated with vehicle (1/1000 EtOH, 1/1000 DMSO). After further incubation of the cells for 48 hrs, growth medium was removed and cells were fixed in trichloroacetic acid (TCA) at a final concentration of 10%. Following incubation of the cells for one hour at 4°C, TCA was removed, plates was washed four times with ddH_20, excess ddH_20 was removed and plate was allowed to dry at room temperature. After TCA-fixation cells were stained with 0.4 % SRB in 1% acetic acid, for 30 minutes. Subsequently, cells were washed with 1% acetic acid, for 4 times, and then the plates was allowed to air-dried. Bound-dye became soluble in 10 mM Tris-Base, upon incubation for 10 minutes, at 37 ^oC, followed by shaking on a shaker, for 2 minutes. Absorbance was measured at 540 nm in a microplate reader (BIO TEK Instruments, EL 311, Winooski, Vermont, United States). Statistical analysis of the results with one way ANOVA followed by Tukeys' post-hoc test showed no statistical significant effect ($F=1.2$; $F_{crit}=2$; $DF_{between\ groups}=11$; $DF_{within\ groups}=39$; $DF_{total}=50$, $P=0.34$)

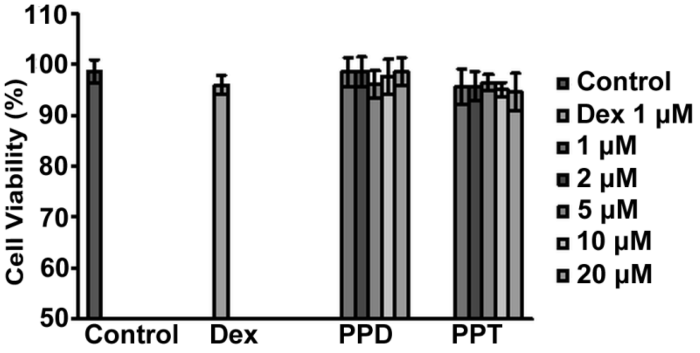

Figure S2. Effect of PPD and PPT on HeLa cell growth inhibition. Cell viability was determined by SRB assay and expressed as % viability compared to control vehicle treated cells. Results are expressed as means ± SD, n>3. No statistically significant effect of PPD and PPT on cells viability was observed.

3. Quantification of the effect of the PPD and PPT compound on PEPCK and GR protein levels in HepG2 cells

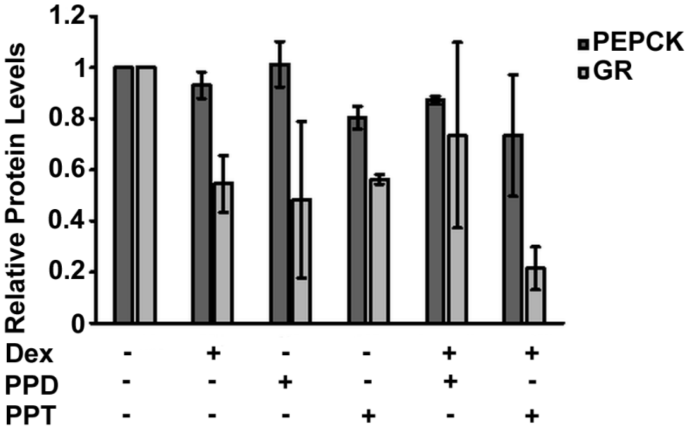

Figure S3. Quantification of the results from Western blot analysis of GR, PEPCK and β-actin protein levels in HepG2 cells treated with 10 μM of PPD or PPT in combination or not with 1 μM DEX. Relative protein levels indicate the mean ± S.D. of ratios resulting from normalization of PEPCK or GR protein levels against β-actin from two independent experiments. Quantifications of protein bands was performed by ImageJ v1.47 program (NIH, Bethesda, MD, USA)

4. Full statistics - Details on the Statistical analysis of the results

Statistical report is followed

Full Statistics

Figure 3 B

**ANOVA**							
**Source of Variation**	**d.f.**	**SS**	**MS**	**F**	**p-value**	**F crit**	**Omega Sqr.**
**Between Groups**	3	4,77875	1,59292	143,28126	0,	2,6903	0,7951
**Within Groups**	106	1,17845	0,01112				
**Total**	109	5,9572					
**Hartley Fmax**	6,31005	Degrees of Freedom	4	29			
**Cochran C**	0,36748	Degrees of Freedom	4	29			
**Bartlett Chi-square**	22,70227	Degrees of Freedom	3	*p-value*	0,00005		

Comparisons among groups (Factor 1-Factor)

**Tukey-Kramer**				
**Groups**	**Difference**	**Test Statistic**	**p-value**	**Significant**
**Control vs DEX**	-0,54569	28,10569	-0,00002	*Yes*
**Control vs PPD**	-0,13853	6,48474	0,00005	*Yes*
**Control vs PPT**	-0,3048	15,69847	-0,00002	*Yes*
**DEX vs PPD**	0,40716	19,19381	-0,00002	*Yes*
**DEX vs PPT**	0,24089	12,51372	-0,00002	*Yes*
**PPD vs PPT**	-0,16626	7,8378	-0,00002	*Yes*

Figure 5 A

**Summary**	
**Response**	GR Transcriptional Activity
**Factor #1**	Triterpene
**Factor #2**	DEX

Factor	Group
Triterpene	Control
Triterpene	PPD
Triterpene	PPT
DEX	+
DEX	-
Triterpene x DEX	Control x +
Triterpene x DEX	Control x -
Triterpene x DEX	PPD x +
Triterpene x DEX	PPD x -
Triterpene x DEX	PPT x +
Triterpene x DEX	PPT x -

**ANOVA**							
**Source of Variation**	**SS**	**d.f.**	**MS**	**F**	**p-value**	**F crit**	**Omega Sqr.**
**Factor #1 (Triterpene)**	9,16673	2	4,58337	1,0543	0,35354	3,11864	0,00048
**Factor #2 (DEX)**	588,02917	1	588,02917	135,26332	0,	3,96847	0,5977
**Factor #1 + #2 (Triterpene x DEX)**	48,9615	2	24,48075	5,63126	0,00527	3,11864	0,04123
**Within Groups**	326,04691	75	4,34729				
**Total**	972,20431	80	12,15255				
**Omega squared for combined effect**	0,63941						

1) Comparisons among groups of factor 2 (DEX) within each factor 1 (Triterpene) level

a) Factor 1 (Triterpene) = Control

**Tukey-Kramer**				
**Groups**	**Difference**	**Test Statistic**	**p-value**	**Significant**
**+ vs -**	7,51981	11,56684	0,00007	Yes

b) Factor 1 (Triterpene) = PPD

**Tukey-Kramer**				
**Groups**	**Difference**	**Test Statistic**	**p-value**	**Significant**
**+ vs -**	5,01522	9,12771	0,00007	Yes

c) Factor 1 (Triterpene) = PPT

**Tukey-Kramer**				
**Groups**	**Difference**	**Test Statistic**	**p-value**	**Significant**
+ vs -	4,78825	8,71463	0,00007	Yes

2) Comparisons among groups of factor 1 (Triterpene) within each factor 2 (DEX) level

a) Factor 2 (DEX) = +

**Tukey-Kramer**				
**Groups**	**Difference**	**Test Statistic**	**p-value**	**Significant**
**Control vs PPD**	2,95428	4,90832	0,00249	Yes
**Control vs PPT**	2,32748	3,86693	0,02102	Yes
**PPD vs PPT**	-0,6268	1,27544	0,64082	No

b) Factor 2 (DEX) = -

**Tukey-Kramer**				
**Groups**	**Difference**	**Test Statistic**	**p-value**	**Significant**
**Control vs PPD**	0,44969	0,74713	0,85774	No
**Control vs PPT**	-0,40408	0,67136	0,88342	No
**PPD vs PPT**	-0,85377	1,41848	0,57729	No

Figure 6

**Summary**	
**Response**	NFkbeta Activity
**Factor #1**	TNF
**Factor #2**	Treatment

**Factor**	**Group**
TNF	+
TNF	-
Treatment	Control
Treatment	DEX
Treatment	PPD
Treatment	PPT
TNF x Treatment	+ x Control
TNF x Treatment	+ x DEX
TNF x Treatment	+ x PPD
TNF x Treatment	+ x PPT
TNF x Treatment	- x Control
TNF x Treatment	- x DEX
TNF x Treatment	- x PPD
TNF x Treatment	- x PPT

Figure 6 A

ANOVA							
**Source of Variation**	**SS**	**d.f.**	**MS**	**F**	**p-value**	**F crit**	**Omega Sqr.**
**Factor #1 (TNF)**	12,17724	1	12,17724	214,3615	1,09806E-10	4,494	0,45263
**Factor #2 (Treatment)**	7,62998	3	2,54333	44,77132	5,23506E-8	3,23887	0,27857
**Factor #1 + #2 (TNF x Treatment)**	6,00491	3	2,00164	35,2357	2,79888E-7	3,23887	0,21788
**Within Groups**	0,90891	16	0,05681				
**Total**	26,72104	23	1,16178				
**Omega squared for combined effect**	0,94909						

1) Comparisons among groups (Factor 2 - Treatment)

**Tukey B**				
**Groups**	**Difference**	**Test Statistic**	**p-value**	**Significant**
**DEX vs PPT (df = 2)**	-0,10382	1,06697	0,66747	No
**PPD vs PPT (df = 3)**	-0,51323	5,27461	0,00691	Yes
**Control vs PPT (df = 4)**	-1,4301	14,69741	0,00003	Yes
**DEX vs PPD (df = 2)**	-0,40942	4,20764	0,02454	Yes
**Control vs DEX (df = 3)**	-1,32628	13,63044	0,00005	Yes
**Control vs PPD (df = 2)**	-0,91686	9,4228	0,00008	Yes

2) Comparisons among groups of factor 2 (Treatment) within each factor 1 (TNF) level

a) Factor 1 (TNF) = +

**Tukey B**				
**Groups**	**Difference**	**Test Statistic**	**p-value**	**Significant**
**DEX vs PPT (df = 2)**	-0,39553	2,87432	0,13827	No
**PPD vs PPT (df = 3)**	-0,86522	6,28762	0,00159	Yes
**Control vs PPT (df = 4)**	-2,75818	20,04392	0,00003	Yes
**DEX vs PPD (df = 2)**	-0,46969	3,4133	0,07134	No
**Control vs DEX (df = 3)**	-2,36266	17,1696	0,00005	Yes
**Control vs PPD (df = 2)**	-1,89296	13,7563	0,00007	Yes

b) Factor 1 (TNF) = -

**Tukey B**				
**Groups**	Difference	Test Statistic	p-value	Significant
**DEX vs PPT (df = 2)**	-0,18789	1,3654	0,55965	No
**Control vs DEX (df = 3)**	-0,2899	2,10675	0,39395	No
**DEX vs PPD (df = 4)**	-0,34914	2,53721	0,31181	No
**Control vs PPT (df = 2)**	-0,10202	0,74135	0,77963	No
**PPD vs PPT (df = 3)**	-0,16125	1,17181	0,76573	No
**Control vs PPD (df = 2)**	-0,05923	0,43045	0,8773	No

3) Comparisons among groups of factor 1 (TNF) within each factor 2 (Treatment) level

a) Factor 2 (Treatment) = Control

**Tukey B**				
**Groups**	**Difference**	**Test Statistic**	**p-value**	**Significant**
+ vs - (df = 2)	-3,0949	22,49086	0,00011	Yes

b) Factor 2 (Treatment) = DEX

**Tukey B**				
**Groups**	**Difference**	**Test Statistic**	**p-value**	**Significant**
+ vs - (df = 2)	-1,02215	7,42801	0,00018	Yes

c) Factor 2 (Treatment) = PPD

**Tukey B**				
**Groups**	**Difference**	**Test Statistic**	**p-value**	**Significant**
+ vs - (df = 2)	-1,1427	8,3041	0,00013	Yes

d) Factor 2 (Treatment) = PPT

**Tukey B**				
**Groups**	**Difference**	**Test Statistic**	**p-value**	**Significant**
+ vs - (df = 2)	-0,43873	3,18829	0,03856	Yes

Figure 6 B

**ANOVA**							
**Source of Variation**	**SS**	**d.f.**	**MS**	**F**	**p-value**	**F crit**	**Omega Sqr.**
**Factor #1 (TNF)**	13,48348	1	13,48348	78,06254	0,	3,94454	0,37115
**Factor #2 (Treatment)**	0,69183	3	0,23061	1,33511	0,26782	2,70359	0,00484
**Factor #1 + #2 (TNF x Treatment)**	5,62435	3	1,87478	10,85404	3,58932E-6	2,70359	0,14238
**Within Groups**	15,89085	92	0,17273				
**Total**	35,69051	99	0,36051				
**Omega squared for combined effect**	0,51837						

1) Comparisons among groups of factor 2 (Treatment) within each factor 1 (TNF) level

a) Factor 1 (TNF) = +

**Tukey Kramer**				
**Groups**	**Difference**	**Test Statistic**	**p-value**	**Significant**
**Control vs Dex**	0,85808	4,61671	0,00828	Yes
**Control vs PPD**	0,87068	4,50073	0,0106	Yes
**Control vs PPT**	1,40078	7,44178	-0,00001	Yes
**Dex vs PPD**	0,0126	0,10503	0,99987	No
**Dex vs PPT**	0,5427	4,87341	0,00471	Yes
**PPD vs PPT**	0,5301	4,28844	0,01643	Yes

b) Factor 1 (TNF) = -

**Tukey-Kramer**				
**Groups**	**Difference**	**Test Statistic**	**p-value**	**Significant**
**Control vs Dex**	0,3021	2,90191	0,17682	No
**Control vs PPD**	0,22013	2,11452	0,44458	No
**Control vs PPT**	0,14545	1,3127	0,78985	No
**Dex vs PPD**	-0,08197	0,76387	0,94903	No
**Dex vs PPT**	-0,15665	1,37633	0,76504	No
**PPD vs PPT**	-0,07468	0,65615	0,96677	No

2) Comparisons among groups of factor 1 (TNF) within each factor 2 (Treatment) level

a) Factor 2 (Treatment) = Control

Tukey Kramer				
**Groups**	**Difference**	**Test Statistic**	**p-value**	**Significant**
+ vs -	1,55468	8,44784	0,00006	Yes

b) Factor 2 (Treatment) = DEX

Tukey Kramer				
**Groups**	**Difference**	**Test Statistic**	**p-value**	**Significant**
+ vs -	0,9987	9,30683	0,00006	Yes

c) Factor 2 (Treatment) = PPD

Tukey Kramer				
**Groups**	**Difference**	**Test Statistic**	**p-value**	**Significant**
+ vs -	0,90413	7,53602	0,00006	Yes

d) Factor 2 (Treatment) = PPT

Tukey Kramer				
**Groups**	**Difference**	**Test Statistic**	**p-value**	**Significant**
+ vs -	0,29935	2,54454	0,0753	No

Figure 6 C

ANOVA							
**Source of Variation**	**SS**	**d.f.**	**MS**	**F**	**p-value**	**F crit**	**Omega Sqr.**
**Factor #1 (TNF)**	757,85656	1	757,85656	276,43168	1,61694E-11	4,494	0,86076
**Factor #2 (Treatment)**	36,3362	3	12,11207	4,41793	0,01914	3,23887	0,03204
**Factor #1 + #2 (TNF x Treatment)**	36,46459	3	12,15486	4,43354	0,01891	3,23887	0,03219
**Within Groups**	43,86511	16	2,74157				
**Total**	874,52246	23	38,02272				
**Omega squared for combined effect**	0,925						

1) Comparisons among groups (Factor 2 - Treatment)

Tukey B				
**Groups**	**Difference**	**Test Statistic**	**p-value**	**Significant**
**DEX vs PPT (df = 2)**	-0,86584	1,28089	0,59024	No
**DEX vs PPD (df = 3)**	-2,32366	3,43755	0,08879	No
**Control vs DEX (df = 4)**	-3,16012	4,67498	0,02093	Yes
**PPD vs PPT (df = 2)**	-1,45783	2,15666	0,29671	No
**Control vs PPT (df = 3)**	-2,29429	3,39409	0,09388	No
**Control vs PPD (df = 2)**	-0,83646	1,23743	0,60603	No

2) Comparisons among groups of factor 2 (Treatment) within each factor 1 (TNF) level

a) Factor 1 (TNF) = +

Tukey B				
**Groups**	**Difference**	**Test Statistic**	**p-value**	**Significant**
**DEX vs PPT (df = 2)**	-1,63557	1,71093	0,43691	No
**DEX vs PPD (df = 3)**	-4,46083	4,66634	0,01657	Yes
**Control vs DEX (df = 4)**	-6,36043	6,65346	0,00121	Yes
**PPD vs PPT (df = 2)**	-2,82525	2,95542	0,12567	No
**Control vs PPT (df = 3)**	-4,72486	4,94253	0,01115	Yes
**Control vs PPD (df = 2)**	-1,8996	1,98712	0,34674	No

b) Factor 1 (TNF) = -

Tukey B				
Groups	Difference	Test Statistic	p-value	Significant
**Control vs DEX (df = 2)**	-0,04018	0,04204	0,98838	No
**Control vs PPT (df = 3)**	-0,13628	0,14256	0,99707	No
**Control vs PPD (df = 4)**	-0,22668	0,23713	0,99832	No
**DEX vs PPT (df = 2)**	-0,0961	0,10053	0,97206	No
**DEX vs PPD (df = 3)**	-0,1865	0,19509	0,99437	No
**PPD vs PPT (df = 2)**	-0,0904	0,09456	0,97372	No

3) Comparisons among groups of factor 1 (TNF) within each factor 2 (Treatment) level

a) Factor 2 (Treatment) = Control

Tukey B				
Groups	Difference	Test Statistic	p-value	Significant
+ vs - (df = 2)	-14,58576	15,25773	0,00011	Yes

b) Factor 2 (Treatment) = DEX

Tukey B				
Groups	Difference	Test Statistic	p-value	Significant
+ vs - (df = 2)	-8,18514	8,56224	0,00012	Yes

c) Factor 2 (Treatment) = PPD

Tukey B				
Groups	Difference	Test Statistic	p-value	Significant
+ vs - (df = 2)	-12,45947	13,03349	0,00011	Yes

d) Factor 2 (Treatment) = PPT

Tukey B				
Groups	Difference	Test Statistic	p-value	Significant
+ vs - (df = 2)	-9,72462	10,17264	0,00011	Yes

Figure 6 D

ANOVA							
**Source of Variation**	**SS**	**d.f.**	**MS**	**F**	**p-value**	**F crit**	**Omega Sqr.**
**Factor #1 (TNF)**	77,25888	1	77,25888	87,40489	1,20238E-7	4,54308	0,77352
**Factor #2 (Treatment)**	2,11304	3	0,70435	0,79684	0,5146	3,28738	0,
**Factor #1 + #2 (TNF x Treatment)**	5,22244	3	1,74081	1,96943	0,16194	3,28738	0,02604
**Within Groups**	13,25879	15	0,88392				
**Total**	97,85315	22	4,44787				
**Omega squared for combined effect**	0,7941						

1) Comparisons among groups of factor 2 (Treatment) within each factor 1 (TNF) level

a) Factor 1 (TNF) = +

Tukey Kramer				
**Groups**	**Difference**	**Test Statistic**	**p-value**	**Significant**
**Control vs Dex**	-0,53714	0,98955	0,89566	No
**Control vs PPD**	-1,47805	2,72297	0,2592	No
**Control vs PPT**	0,02409	0,0397	0,99999	No
**Dex vs PPD**	-0,94091	1,73341	0,62095	No
**Dex vs PPT**	0,56123	0,92478	0,91261	No
**PPD vs PPT**	1,50214	2,4752	0,33389	No

b) Factor 1 (TNF) = -

Tukey-Kramer				
**Groups**	**Difference**	**Test Statistic**	**p-value**	**Significant**
**Control vs Dex**	0,10703	0,19718	0,99905	No
**Control vs PPD**	-0,14174	0,26113	0,99773	No
**Control vs PPT**	-1,18371	2,18073	0,43879	No
**Dex vs PPD**	-0,24877	0,4583	0,98782	No
**Dex vs PPT**	-1,29074	2,3779	0,36673	No
**PPD vs PPT**	-1,04197	1,9196	0,54314	No

2) Comparisons among groups of factor 1 (TNF) within each factor 2 (Treatment) level

a) Factor 2 (Treatment) = Control

Tukey Kramer				
**Groups**	**Difference**	**Test Statistic**	**p-value**	**Significant**
+ vs -	3,42842	6,31609	0,00055	Yes

b) Factor 2 (Treatment) = DEX

Tukey Kramer				
**Groups**	**Difference**	**Test Statistic**	**p-value**	**Significant**
+ vs -	4,07259	7,50282	0,00019	Yes

c) Factor 2 (Treatment) = PPD

Tukey Kramer				
**Groups**	**Difference**	**Test Statistic**	**p-value**	**Significant**
+ vs -	4,76473	8,77793	0,00013	Yes

d) Factor 2 (Treatment) = PPT

Tukey Kramer				
**Groups**	**Difference**	**Test Statistic**	**p-value**	**Significant**
+ vs -	2,22061	3,65908	0,02063	Yes

Figure S1

1) **Comparison among Control and Dex**

**ANOVA**							
**Source of Variation**	**d.f.**	**SS**	**MS**	**F**	**p-value**	**F crit**	**Omega Sqr.**
**Between Groups**	1	2,98605	2,98605	442,45748	0,	4,06705	0,90749
**Within Groups**	43	0,2902	0,00675				
**Total**	44	3,27625					
**Hartley Fmax**	1,95256	Degrees of Freedom	2	28			
**Cochran C**	0,66131	Degrees of Freedom	2	28			
**Bartlett Chi-square**	2,2408	Degrees of Freedom	1	p-value	0,13441		

Comparisons among groups (Control-Dex)

**Tukey B**				
Groups	**Difference**	**Test Statistic**	**p-value**	**Significant**
**Control vs Dex (df = 2)**	-0,53814	26,20252	0,00007	*Yes*

2) **Comparison among group, which indicated in the table**

**Groups**
Control
Dex
PPD 0,1 μM
PPD 1 μM
PPD 2 μM
PPD 10 μM
PPD 20 μM
PPT 0,1 μM
PPT 0,5 μM
PPT 1 μM
PPT 2 μM
PPT 5 μM
PPT 10 μM
PPT 20 μM

ANOVA							
**Source of Variation**	**d.f.**	**SS**	**MS**	**F**	**p-value**	**F crit**	**Omega Sqr.**
**Between Groups**	13	3,57358	0,27489	40,80358	0,	1,78461	0,75601
**Within Groups**	153	1,03075	0,00674				
**Total**	166	4,60433					
**Hartley Fmax**	8,4545	Degrees of Freedom	14	28			
**Cochran C**	0,13879	Degrees of Freedom	14	28			
**Bartlett Chi-square**	17,46755	Degrees of Freedom	13	p-value	0,1788		

Tukey-Kramer				
**Groups**	**Difference**	**Test Statistic**	**p-value**	**Significant**
**Control vs Dex**	-0,53814	29,7737	0,74636	*No*
**Control vs PPD 0,1 μM**	-0,41141	18,5621	0,74636	*No*
**Control vs PPD 1 μM**	-0,44223	20,82078	0,74636	*No*
**Control vs PPD 10 μM**	-0,50741	25,44497	0,74636	*No*
**Control vs PPD 2 μM**	-0,38526	15,9311	0,74636	*No*
**Control vs PPD 20 μM**	-0,44072	18,22457	0,74636	*No*
**Control vs PPT 0,1 μM**	-0,34699	13,19316	0,74636	*No*
**Control vs PPT 0,5 μM**	-0,30705	10,32582	0,74636	*No*
**Control vs PPT 1 μM**	-0,33095	14,14551	0,74636	*No*
**Control vs PPT 10 μM**	-0,44886	17,8605	0,74636	*No*
**Control vs PPT 2 μM**	-0,39082	17,63333	0,74636	*No*
**Control vs PPT 20 μM**	-0,48145	20,57805	0,74636	*No*
**Control vs PPT 5 μM**	-0,44035	17,52203	0,74636	*No*
**Dex vs PPD 0,1 μM**	0,12673	6,36173	0,74651	*No*
**Dex vs PPD 1 μM**	0,09591	5,07779	0,75214	*No*
**Dex vs PPD 10 μM**	0,03073	1,76445	0,99842	*No*
**Dex vs PPD 2 μM**	0,15288	6,90358	0,74639	*No*
**Dex vs PPD 20 μM**	0,09742	4,39914	0,77214	*No*
**Dex vs PPT 0,1 μM**	0,19115	7,82082	0,74637	*No*
**Dex vs PPT 0,5 μM**	0,23109	8,22274	0,74636	*No*
**Dex vs PPT 1 μM**	0,20719	9,73471	0,74636	*No*

**Dex vs PPT 10 μM**	0,08928	3,85203	0,81342	*No*
**Dex vs PPT 2 μM**	0,14732	7,39505	0,74637	*No*
**Dex vs PPT 20 μM**	0,0567	2,6638	0,95837	*No*
**Dex vs PPT 5 μM**	0,09779	4,21903	0,78281	*No*
**PPD 0,1 μM vs PPT 1 μM**	-0,03082	1,35005	0,9999	*No*
**PPD 0,1 μM vs PPD 10 μM**	-0,09601	4,43862	0,77042	*No*
**PPD 0,1 μM vs PPD 2 μM**	0,02615	1,02177	1,	*No*
**PPD 0,1 μM vs PPD 20 μM**	-0,02931	1,14535	0,99998	*No*
**PPD 0,1 μM vs PPT 0,1 μ M**	0,06441	2,33363	0,98293	*No*
**PPD 0,1 μM vs PPT 0,5 μM**	0,10436	3,37806	0,87028	*No*
**PPD 0,1 μM vs PPT 1 μM**	0,08046	3,23765	0,88915	*No*
**PPD 0,1 μM vs PPT 10 μM**	-0,03745	1,41379	0,99983	*No*
**PPD 0,1 μM vs PPT 2 μM**	0,02059	0,86878	1,	*No*
**PPD 0,1 μM vs PPT 20 μM**	-0,07004	2,81838	0,94233	*No*
**PPD 0,1 μM vs PPT 5 μM**	-0,02895	1,09269	0,99999	*No*
**PPD 1 μM vs PPD 10 μM**	-0,06518	3,1516	0,90071	*No*
**PPD 1 μM vs PPD 2 μM**	0,05697	2,29765	0,98479	*No*
**PPD 1 μM vs PPD 20 μM**	0,00151	0,06098	1,	*No*
**PPD 1 μM vs PPT 0,1 μ M**	0,09524	3,5449	0,84864	*No*
**PPD 1 μM vs PPT 0,5 μM**	0,13518	4,47076	0,76892	*No*
**PPD 1 μM vs PPT 1 μM**	0,11128	4,63092	0,76251	*No*
**PPD 1 μM vs PPT 10 μM**	-0,00663	0,25766	1,	*No*
**PPD 1 μM vs PPT 2 μM**	0,05141	2,25163	0,98696	*No*
**PPD 1 μM vs PPT 20 μM**	-0,03921	1,63186	0,99926	*No*
**PPD 1 μM vs PPT 5 μM**	0,00188	0,07302	1,	*No*
**PPD 10 μM vs PPD 2 μM**	0,12216	5,15552	0,75112	*No*
**PPD 10 μM vs PPD 20 μM**	0,06669	2,81476	0,94273	*No*
**PPD 10 μM vs PPT 0,1 μM**	0,16042	6,20525	0,74661	*No*
**PPD 10 μM vs PPT 0,5 μM**	0,20037	6,8291	0,74639	*No*
**PPD 10 μM vs PPT 1 μM**	0,17646	7,70896	0,74637	*No*
**PPD10 μM vs PPT 10 μM**	0,05855	2,37427	0,98064	*No*
**PPD 10 μM vs PPT 2 μM**	0,11659	5,39032	0,74891	*No*
**PPD 10 μM vs PPT 20 μM**	0,02597	1,13441	0,99999	*No*
**PPD 10 μM vs PPT 5 μM**	0,06706	2,71919	0,95293	*No*
**PPD 2 μM vs PPD 20 μM**	-0,05546	2,02716	0,99445	*No*
**PPD 2 μM vs PPT 0,1 μ M**	0,03826	1,30826	0,99993	*No*
**PPD 2 μM vs PPT 0,5 μM**	0,07821	2,41595	0,97808	*No*
**PPD 2 μM vs PPT 1 μM**	0,05431	2,03653	0,99423	*No*
**PPD 2 μM vs PPT 10 μM**	-0,0636	2,25527	0,9868	*No*
**PPD 2 μM vs PPT 2 μM**	-0,00556	0,21744	1,	*No*
**PPD 2 μM vs PPT 20 μM**	-0,09619	3,60703	0,84092	*No*

**PPD 2 μM vs PPT 5 μM**	-0,0551	1,95364	0,99597	*No*
**PPD 20 μM vs PPT 0,1 μ M**	0,09373	3,20449	0,89361	*No*
**PPD 20 μM vs PPT 0,5 μM**	0,13367	4,12921	0,78917	*No*
**PPD 20 μM vs PPT 1 μM**	0,10977	4,11635	0,79014	*No*
**PPD 20 μM vs PPT 10 μM**	-0,00814	0,28864	1,	*No*
**PPD 20 μM vs PPT 2 μM**	0,0499	1,94969	0,99605	*No*
**PPD 20 μM vs PPT 20 μM**	-0,04073	1,52721	0,99962	*No*
**PPD 20 μM vs PPT 5 μM**	0,00037	0,01299	1,	*No*
**PPT 0,1 μ M vs PPT 0,5 μM**	0,03994	1,17541	0,99998	*No*
**PPT 0,1 μ M vs PPT 1 μM**	0,01604	0,56091	1,	*No*
**PPT 0,1 μ M vs PPT 10 μM**	-0,10187	3,3913	0,86852	*No*
**PPT 0,1 μ M vs PPT 2 μM**	-0,04383	1,58787	0,99944	*No*
**PPT 0,1 μ M vs PPT 20 μM**	-0,13445	4,70088	0,76022	*No*
**PPT 0,1 μ M vs PPT 5 μM**	-0,09336	3,10812	0,90649	*No*
**PPT 0,5 μM vs PPT 1 μM**	-0,0239	0,7519	1,	*No*
**PPT 0,5 μM vs PPT 10 μM**	-0,14181	4,28603	0,77852	*No*
**PPT 0,5 μM vs PPT 2 μM**	-0,08377	2,71173	0,95368	*No*
**PPT 0,5 μM vs PPT 20 μM**	-0,1744	5,48611	0,7483	*No*
**PPT 0,5 μM vs PPT 5 μM**	-0,13331	4,02894	0,79716	*No*
**PPT 1 μM vs PPT 10 μM**	-0,11791	4,28295	0,77871	*No*
**PPT 1 μM vs PPT 2 μM**	-0,05987	2,4093	0,9785	*No*
**PPT 1 μM vs PPT 20 μM**	-0,1505	5,79821	0,74707	*No*
**PPT t 1 μM vs PPT 5 μM**	-0,1094	3,97397	0,80194	*No*
**PPT 10 μM vs PPT 2 μM**	0,05804	2,19085	0,98947	*No*
**PPT 10 μM vs PPT 20 μM**	-0,03259	1,18365	0,99998	*No*
**PPT 10 μM vs PPT 5 μM**	0,00851	0,29312	1,	*No*
**PPT 2 μM vs PPT 20 μM**	-0,09062	3,64673	0,83614	*No*
**PPT 2 μM vs PPT 5 μM**	-0,04953	1,86975	0,99729	*No*
**PPT 20 μM vs PPT 5 μM**	0,04109	1,49263	0,9997	*No*

Fisher LSD				
**Group vs. Group (Contrast)**	**Difference**	**Test Statistic**	**p-value**	**Significant**
**Control vs Dex**	-0,53814	21,05318	0,	Yes
**Control vs PPD 0,1 µM**	-0,41141	13,12538	0,	Yes
**Control vs PPD 1 µM**	-0,44223	14,72251	0,	Yes
**Control vs PPD 10 µM**	-0,50741	17,99231	0,	Yes
**Control vs PPD 2 µM**	-0,38526	11,26499	0,	Yes
**Control vs PPD 20 µM**	-0,44072	12,88672	0,	Yes
**Control vs PPT 0,1 µM**	-0,34699	9,32897	0,	Yes
**Control vs PPT 0,5 µM**	-0,30705	7,30146	1,39566E-11	Yes
**Control vs PPT 1 µM**	-0,33095	10,00239	0,	Yes
**Control vs PPT 10 µM**	-0,44886	12,62928	0,	Yes
**Control vs PPT 2 µM**	-0,39082	12,46865	0,	Yes
**Control vs PPT 20 µM**	-0,48145	14,55088	0,	Yes
**Control vs PPT 5 µM**	-0,44035	12,38994	0,	Yes
**Dex vs PPD 0,1 µM**	0,12673	4,49842	0,00001	Yes
**Dex vs PPD 1 µM**	0,09591	3,59054	0,00044	Yes
**Dex vs PPD 10 µ M**	0,03073	1,24766	0,21404	No
**Dex vs PPD 2 µM**	0,15288	4,88157	2,59373E-6	Yes
**Dex vs PPD 20 µM**	0,09742	3,11066	0,00222	Yes
**Dex vs PPT 0,1 µM**	0,19115	5,53016	1,3293E-7	Yes
**Dex vs PPT 0,5 µM**	0,23109	5,81436	3,36555E-8	Yes
**Dex vs PPT 1 µM**	0,20719	6,88348	1,36479E-10	Yes
**Dex vs PPT 10 µM**	0,08928	2,7238	0,00719	Yes
**Dex vs PPT 2 µM**	0,14732	5,22909	5,43682E-7	Yes
**Dex vs PPT 20 µM**	0,0567	1,88359	0,06149	No
**Dex vs PPT 5 µM**	0,09779	2,9833	0,00331	Yes
**PPD 0,1 µM vs PPD 1 µM**	-0,03082	0,95463	0,34125	No
**PPD 0,1 µM vs PPD 10 µM**	-0,09601	3,13858	0,00203	Yes
**PPD 0,1 µM vs PPD 2 µM**	0,02615	0,7225	0,47108	No
**PPD 0,1 µM vs PPD 20 µM**	-0,02931	0,80989	0,41925	No
**PPD 0,1 µM vs PPT 0,1 µ M**	0,06441	1,65012	0,10094	No
**PPD 0,1 µM vs PPT 0,5 µM**	0,10436	2,38865	0,01811	Yes
**PPD 0,1 µM vs PPT 1 µM**	0,08046	2,28936	0,02341	Yes
**PPD 0,1 µM vs PPT 10 µM**	-0,03745	0,9997	0,31901	No
**PPD 0,1 µM vs PPT 2 µM**	0,02059	0,61432	0,5399	No
**PPD 0,1 µM vs PPT 20 µM**	-0,07004	1,9929	0,04803	No
**PPD 0,1 µM vs PPT 5 µM**	-0,02895	0,77265	0,44091	No
**PPD 1 µM vs PPD 10 µM**	-0,06518	2,22852	0,02728	No
**PPD 1 µM vs PPD 2 µM**	0,05697	1,62469	0,10626	No
**PPD 1 µM vs PPD 20 µM**	0,00151	0,04312	0,96566	No

**PPD 1 μM vs PPT 0,1 μM**	0,09524	2,50662	0,01322	Yes
**PPD 1 μM vs PPT 0,5 μM**	0,13518	3,16131	0,00189	Yes
**PPD 1 μM vs PPT 1 μM**	0,11128	3,27456	0,00131	Yes
**PPD 1 μM vs PPT 10 μM**	-0,00663	0,1822	0,85567	No
**PPD 1 μM vs PPT 2 μM**	0,05141	1,59214	0,11339	No
**PPD 1 μM vs PPT 20 μM**	-0,03921	1,1539	0,25032	No
**PPD 1 μM vs PPT 5 μM**	0,00188	0,05164	0,95889	No
**PPD 10 μM vs PPD 2 μM**	0,12216	3,64551	0,00036	Yes
**PPD 10 μM vs PPD 20 μM**	0,06669	1,99034	0,04831	No
**PPD 10 μM vs PPT 0,1 μM**	0,16042	4,38777	0,00002	Yes
**PPD 10 μM vs PPT 0,5 μM**	0,20037	4,82891	3,26635E-6	Yes
**PPD 10 μM vs PPT 1 μM**	0,17646	5,45106	1,93379E-7	Yes
**PPD 10 μM vs PPT 10 μM**	0,05855	1,67886	0,09519	No
**PPD 10 μM vs PPT 2 μM**	0,11659	3,81153	0,0002	Yes
**PPD 10 μM vs PPT 20 μM**	0,02597	0,80215	0,4237	No
**PPD 10 μM vs PPT 5 μM**	0,06706	1,92276	0,05634	No
**PPD 2 μM vs PPD 20 μM**	-0,05546	1,43342	0,15375	No
**PPD 2 μM vs PPT 0,1 μM**	0,03826	0,92508	0,35636	No
**PPD 2 μM vs PPT 0,5 μM**	0,07821	1,70833	0,08958	No
**PPD 2 μM vs PPT 1 μM**	0,05431	1,44004	0,15187	No
**PPD 2 μM vs PPT 10 μM**	-0,0636	1,59471	0,11281	No
**PPD 2 μM vs PPT 2 μM**	-0,00556	0,15375	0,87801	No
**PPD 2 μM vs PPT 20 μM**	-0,09619	2,55056	0,01172	Yes
**PPD 2 μM vs PPT 5 μM**	-0,0551	1,38144	0,16913	No
**PPD 20 μM vs PPT 0,1 μM**	0,09373	2,26592	0,02484	Yes
**PPD 20 μM vs PPT 0,5 μM**	0,13367	2,91979	0,00403	Yes
**PPD 20 μM vs PPT 1 μM**	0,10977	2,9107	0,00414	Yes
**PPD 20 μM vs PPT 10 μM**	-0,00814	0,2041	0,83855	No
**PPD 20 μM vs PPT 2 μM**	0,0499	1,37864	0,16999	No
**PPD 20 μM vs PPT 20 μM**	-0,04073	1,0799	0,28186	No
**PPD 20 μM vs PPT 5 μM**	0,00037	0,00918	0,99268	No
**PPT 0,1 μM vs PPT 0,5 μM**	0,03994	0,83114	0,40717	No
**PPT 0,1 μM vs PPT 1 μM**	0,01604	0,39662	0,69219	No
**PPT 0,1 μM vs PPT 10 μM**	-0,10187	2,39801	0,01767	Yes
**PPT 0,1 μM vs PPT 2 μM**	-0,04383	1,12279	0,26326	No
**PPT 0,1 μM vs PPT 20 μM**	-0,13445	3,32403	0,00111	Yes
**PPT 0,1 μM vs PPT 5 μM**	-0,09336	2,19777	0,02945	No
**PPT 0,5 μM vs PPT 1 μM**	-0,0239	0,53167	0,59572	No
**PPT 0,5 μM vs PPT 10 μM**	-0,14181	3,03068	0,00286	Yes
**PPT 0,5 μM vs PPT 2 μM**	-0,08377	1,91748	0,05702	No
**PPT 0,5 μM vs PPT 20 μM**	-0,1744	3,87927	0,00015	Yes

**PPT 0,5 μM vs PPT 5 μM**	-0,13331	2,84889	0,00498	Yes
**PPT 1 μM vs PPT 10 μM**	-0,11791	3,0285	0,00288	Yes
**PPT 1 μM vs PPT 2 μM**	-0,05987	1,70363	0,09045	No
**PPT 1 μM vs PPT 20 μM**	-0,1505	4,09995	0,00007	Yes
**PPT 1 μM vs PPT 5 μM**	-0,1094	2,81002	0,00559	Yes
**PPT 10 μM vs PPT 2 μM**	0,05804	1,54917	0,12338	No
**PPT 10 μM vs PPT 20 μM**	-0,03259	0,83697	0,4039	No
**PPT 10 μM vs PPT 5 μM**	0,00851	0,20727	0,83607	No
**PPT 2 μM vs PPT 20 μM**	-0,09062	2,57863	0,01085	Yes
**PPT 2 μM vs PPT 5 μM**	-0,04953	1,32211	0,18808	No
**PPT 20 μM vs PPT 5 μM**	0,04109	1,05545	0,29286	No

Figure S2

ANOVA							
Source of Variation	d.f.	SS	MS	F	p-value	F crit	Omega Sqr.
Between Groups	11	0,01052	0,00096	1,15475	0,34858	2,04425	0,0323
Within Groups	39	0,03229	0,00083				
Total	50	0,04281					
Hartley Fmax	11,04338	Degrees of Freedom	12	7			
Cochran C	0,23915	Degrees of Freedom	12	7			
Bartlett Chi-square	6,95631	Degrees of Freedom	11	p-value	0,80261		

Comparisons among groups (Factor 1 - Factor)

**Tukey-Kramer**				
**Groups**	**Difference**	**Test Statistic**	**p-value**	**Significant**
**CONTROL vs DEX**	0,02604	1,89016	0,9686	No
**CONTROL vs PPD 1 μM**	0,00076	0,06139	1,	No
**CONTROL vs PPD 10 μM**	0,01004	0,80568	0,99999	No
**CONTROL vs PPD 2 μM**	0,00086	0,06906	1,	No
**CONTROL vs PPD 20 μM**	-0,0001	0,00767	1,	No
**CONTROL vs PPD 5 μM**	0,02486	1,99502	0,95436	No
**CONTROL vs PPT 1 μM**	0,01702	1,36582	0,99769	No
**CONTROL vs PPT 10 μM**	0,03585	2,87743	0,66844	No
**CONTROL vs PPT 2 μM**	0,02897	2,32497	0,88162	No
**CONTROL vs PPT 20 μM**	0,04063	3,26109	0,48955	No
**CONTROL vs PPT 5 μM**	0,02218	1,78017	0,97971	No
**DEX vs PPD 1 μM**	-0,02527	1,62623	0,98992	No

**DEX vs PPD 10 μM**	-0,016	1,02947	0,99983	No
**DEX vs PPD 2 μM**	-0,02518	1,62008	0,99022	No
**DEX vs PPD 20 μM**	-0,02613	1,6816	0,98687	No
**DEX vs PPD 5 μM**	-0,00118	0,07588	1,	No
**DEX vs PPT 1 μM**	-0,00902	0,58036	1,	No
**DEX vs PPT 10 μM**	0,00982	0,63163	1,	No
**DEX vs PPT 2 μM**	0,00293	0,18867	1,	No
**DEX vs PPT 20 μM**	0,0146	0,93924	0,99993	No
**DEX vs PPT 5 μM**	-0,00386	0,24814	1,	No
**PPD 1 μM vs PPD 10 μM**	0,00927	0,64458	1,	No
**PPD 1 μM vs PPD 2 μM**	0,0001	0,00665	1,	No
**PPD 1 μM vs PPD 20 μM**	-0,00086	0,05981	1,	No
**PPD 1 μM vs PPD 5 μM**	0,02409	1,67458	0,98729	No
**PPD 1 μM vs PPT 1 μM**	0,01625	1,12967	0,99959	No
**PPD 1 μM vs PPT 10 μM**	0,03509	2,43877	0,84612	No
**PPD 1 μM vs PPT 2 μM**	0,0282	1,96032	0,95951	No
**PPD 1 μM vs PPT 20 μM**	0,03987	2,77103	0,71611	No
**PPD 1 μM vs PPT 5 μM**	0,02141	1,48851	0,99511	No
**PPD 10 μM vs PPD 2 μM**	-0,00918	0,63793	1,	No
**PPD 10 μM vs PPD 20 μM**	-0,01013	0,70439	1,	No
**PPD 10 μM vs PPD 5 μM**	0,01482	1,03	0,99983	No
**PPD 10 μM vs PPT 1 μM**	0,00698	0,4851	1,	No
**PPD 10 μM vs PPT 10 μM**	0,02581	1,79419	0,97849	No
**PPD 10 μM vs PPT 2 μM**	0,01893	1,31574	0,99834	No
**PPD 10 μM vs PPT 20 μM**	0,03059	2,12645	0,93072	No
**PPD 10 μM vs PPT 5 μM**	0,01214	0,84393	0,99998	No
**PPD 2 μM vs PPD 20 μM**	-0,00096	0,06645	1,	No
**PPD 2 μM vs PPD 5 μM**	0,024	1,66793	0,98768	No
**PPD 2 μM vs PPT 1 μM**	0,01616	1,12303	0,99962	No
**PPD 2 μM vs PPT 10 μM**	0,03499	2,43212	0,84833	No
**PPD 2 μM vs PPT 2 μM**	0,02811	1,95367	0,96044	No
**PPD 2 μM vs PPT 20 μM**	0,03977	2,76438	0,71902	No
**PPD 2 μM vs PPT 5 μM**	0,02132	1,48187	0,99529	No
**PPD 20 μM vs PPD 5 μM**	0,02495	1,73438	0,98334	No
**PPD 20 μM vs PPT 1 μM**	0,01711	1,18948	0,99934	No
**PPD 20 μM vs PPT 10 μM**	0,03595	2,49858	0,82543	No
**PPD 20 μM vs PPT 2 μM**	0,02906	2,02012	0,95037	No
**PPD 20 μM vs PPT 20 μM**	0,04073	2,83083	0,68956	No
**PPD 20 μM vs PPT 5 μM**	0,02228	1,54832	0,99322	No
**PPD 5 μM vs PPT 1 μM**	-0,00784	0,5449	1,	No
**PPD 5 μM vs PPT 10 μM**	0,01099	0,76419	0,99999	No
**PPD 5 μM vs PPT 2 μM**	0,00411	0,28574	1,	No
**PPD 5 μM vs PPT 20 μM**	0,01577	1,09645	0,99969	No
**PPD 5 μM vs PPT 5 μM**	-0,00268	0,18606	1,	No
**PPT 1 μM vs PPT 10 μM**	0,01883	1,30909	0,99841	No
**PPT 1 μM vs PPT 2 μM**	0,01195	0,83064	0,99998	No
**PPT 1 μM vs PPT 20 μM**	0,02361	1,64135	0,98914	No
**PPT 1 μM vs PPT 5 μM**	0,00516	0,35884	1,	No
**PPT 10 μM vs PPT 2 μM**	-0,00688	0,47845	1,	No
**PPT 10 μM vs PPT 20 μM**	0,00478	0,33226	1,	No
**PPT 10 μM vs PPT 5 μM**	-0,01367	0,95026	0,99992	No
**PPT 2 μM vs PPT 20 μM**	0,01166	0,81071	0,99998	No
**PPT 2 μM vs PPT 5 μM**	-0,00679	0,47181	1,	No
